# A Taxonomy of Bacterial Microcompartment Loci Constructed by a Novel Scoring Method

**DOI:** 10.1371/journal.pcbi.1003898

**Published:** 2014-10-23

**Authors:** Seth D. Axen, Onur Erbilgin, Cheryl A. Kerfeld

**Affiliations:** 1 DOE Joint Genome Institute, Walnut Creek, California, United States of America; 2 Department of Plant and Microbial Biology, University of California, Berkeley, Berkeley, California, United States of America; 3 DOE Plant Research Laboratory, Michigan State University, East Lansing, Michigan, United States of America; 4 Physical Biosciences Division, Lawrence Berkeley National Laboratory, Berkeley, California, United States of America; 5 Berkeley Synthetic Biology Institute, Berkeley, California, United States of America; University of New South Wales, Australia

## Abstract

Bacterial microcompartments (BMCs) are proteinaceous organelles involved in both autotrophic and heterotrophic metabolism. All BMCs share homologous shell proteins but differ in their complement of enzymes; these are typically encoded adjacent to shell protein genes in genetic loci, or operons. To enable the identification and prediction of functional (sub)types of BMCs, we developed LoClass, an algorithm that finds putative BMC loci and inventories, weights, and compares their constituent pfam domains to construct a locus similarity network and predict locus (sub)types. In addition to using LoClass to analyze sequences in the Non-redundant Protein Database, we compared predicted BMC loci found in seven candidate bacterial phyla (six from single-cell genomic studies) to the LoClass taxonomy. Together, these analyses resulted in the identification of 23 different types of BMCs encoded in 30 distinct locus (sub)types found in 23 bacterial phyla. These include the two carboxysome types and a divergent set of metabolosomes, BMCs that share a common catalytic core and process distinct substrates via specific signature enzymes. Furthermore, many Candidate BMCs were found that lack one or more core metabolosome components, including one that is predicted to represent an entirely new paradigm for BMC-associated metabolism, joining the carboxysome and metabolosome. By placing these results in a phylogenetic context, we provide a framework for understanding the horizontal transfer of these loci, a starting point for studies aimed at understanding the evolution of BMCs. This comprehensive taxonomy of BMC loci, based on their constituent protein domains, foregrounds the functional diversity of BMCs and provides a reference for interpreting the role of BMC gene clusters encoded in isolate, single cell, and metagenomic data. Many loci encode ancillary functions such as transporters or genes for cofactor assembly; this expanded vocabulary of BMC-related functions should be useful for design of genetic modules for introducing BMCs in bioengineering applications.

## Author Summary

Some enzymatic transformations have undesirable side reactions, produce toxic or volatile intermediates, or are inefficient; these shortcomings can be alleviated through their sequestration with their substrates in a confined space, as in the membrane-bound organelles of eukaryotes. Recently, it was discovered that bacteria also form organelles–bacterial microcompartments (BMCs)–composed of a protein shell that surrounds functionally related enzymes. BMCs long evaded detection because they typically form only in the presence of the substrate they metabolize, and they can only be visualized by electron microscopy. A few BMCs have been experimentally characterized; they have diverse functions in CO_2_ fixation, pathogenesis, and niche colonization. While the encapsulated enzymes differ among functionally distinct BMCs, the shell architecture is conserved. This enables their detection computationally, as genes for shell proteins are typically nearby genes for the encapsulated enzymes. We developed a novel algorithm to comprehensively identify and categorize BMCs in sequenced bacterial genomes. We show that BMCs are often encoded adjacent to genes that play supporting roles to the organelle's function. Our results provide the first glimpse of the extent of BMC metabolic diversity and will inform design of genetic modules encoding BMCs for introduction of new metabolic functions in a plug-and-play approach.

## Introduction

**Figure 1:**
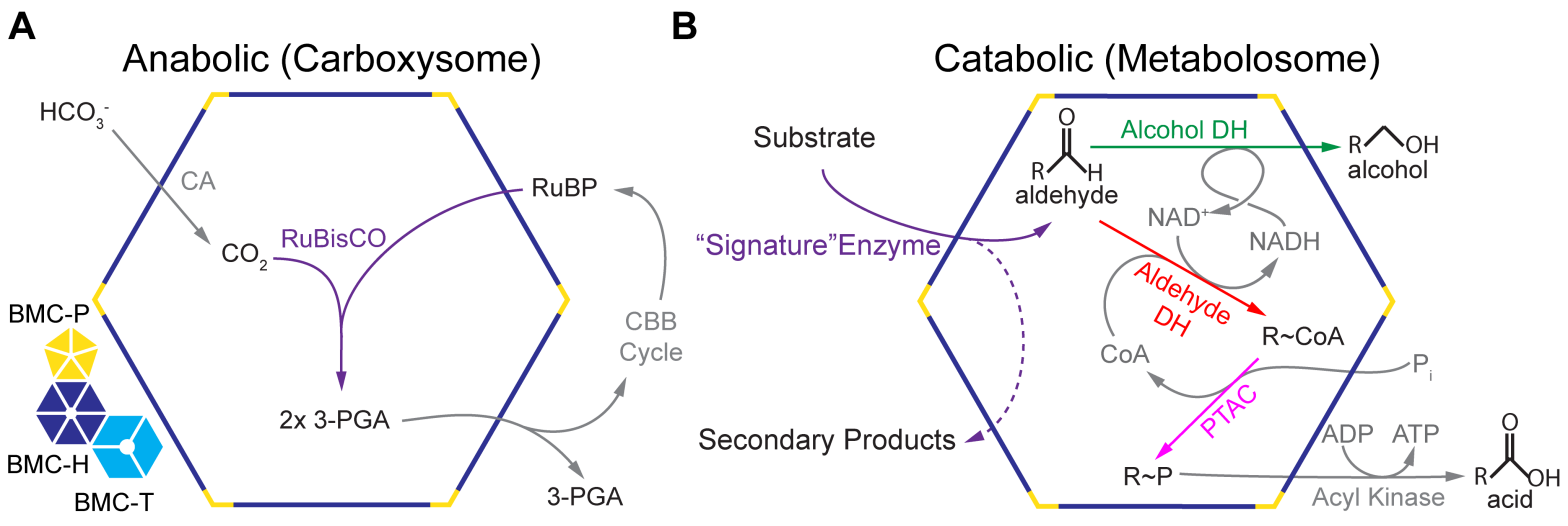
Schematic overview of characterized bacterial microcompartments. (A) Carboxysome. (B) Metabolosome. An example substrate is ethanolamine and the signature enzyme produces acetaldehyde and ammonia, a secondary product. Reactions in gray are peripheral reactions to the core BMC chemistry. BMC shell protein oligomers are depicted on the left: blue, BMC-H; cyan, BMC-T; yellow, BMC-P. 3-PGA, 3-phosphoglycerate, and RuBP, ribulose 1,5-bisphosphate.

Membrane-bound organelles for compartmentalization of specific functions are the hallmark feature of all eukaryotic cells. Bacteria also have organelles, but they are not ubiquitous throughout the domain; instead they are sporadically distributed and frequently provide functions that are key to niche specialization. For example, anammoxosomes are lipid-bound compartments that enable certain planctomycetes to obtain energy from anaerobic ammonium oxidation (reviewed in [1]), and magnetosomes are invaginations of the inner membrane that allow magnetotactic bacteria to orient along the Earth's magnetic field to search for microaerobic environments (reviewed in [2]). Another type of organelle, composed entirely of protein, is the bacterial microcompartment (BMC) (reviewed in [3]–[5]). BMCs were discovered initially in electron micrographs as polyhedral bodies within members of the Cyanobacteria [6] and later in chemoautotrophs [7]. Subsequently, it was shown that these inclusions encapsulate enzymes required for carbon fixation, and they were termed carboxysomes [8]. X-ray crystallographic studies of subunits of the carboxysome shell provided the first structural examples of each of the three main types of BMC shell proteins [9]–[11]. These led to an icosahedral model of the shell: facets formed by (pseudo)hexameric building blocks capped by pentameric vertices (Fig. 1). Hexameric (BMC-H) shell subunits are formed by proteins that contain a single copy of the Pfam [12] domain PF00936, while BMC-T proteins are a fusion of two PF00936 domains and form trimers (pseudohexamers) [10], [13]. Pentagonal vertices are formed by BMC-P proteins, which contain a single PF03319 domain [9], [14], [15]. Several studies have shown that all three components are required for the construction of fully functional carboxysomes [16]–[18].

Carboxysomes were thought to be a peculiarity confined to some autotrophic organisms until operons involved in vitamin B_12_-dependent propanediol (PDU) and ethanolamine utilization (EUT) in *Salmonella enterica* were sequenced and found to encode genes for BMC-H, BMC-T, and BMC-P proteins [19]–[22]. The formation of carboxysome-like structures was subsequently confirmed by electron microscopy of cells grown on 1,2-propanediol or ethanolamine [22], [23]. These studies provided the first examples of catabolic BMCs used by heterotrophic bacteria to degrade specific compounds, in contrast to the anabolic carboxysomes. More recently, the availability of genomic sequence data has enabled the discovery of the prevalence of BMCs among the Bacteria. Shell protein genes are typically found clustered with genes for putative enzymes, allowing prediction of the potential to form BMCs to be made from sequence data. Recently, the first functional characterizations of such bioinformatically-predicted BMCs were reported: one that degrades propanediol via a glycyl radical enzyme (termed the fucosome) [24] and a second that degrades fucose and rhamnose directly via an aldolase (termed the PVM BMC) [25]. For visualization of catabolic BMCs, knowledge of the substrate is necessary in order to induce the operon and organelle formation for subsequent visualization by electron microscopy. This contrasts with historic organelle discovery, when light microscopy of eukaryotic cells was sufficient to reveal new organelles.

Whether anabolic or catabolic, experimentally characterized BMCs share a common functional theme: the shell sequesters enzymes and provides a diffusion barrier for volatile and/or toxic reaction intermediates. For example, in carboxysomes, CO_2_ is generated by carbonic anhydrase (CA) and then fixed within the compartment by RuBisCO; the shell helps to confine the CO_2_ near RuBisCO [26] (Fig. 1A). Likewise, the PDU and EUT BMC shells prevent the leakage of propionaldehyde [27] and acetaldehyde [28], respectively, which are toxic and/or volatile intermediates of the encapsulated biochemical pathway. These experimental observations were used to infer the type of aldehyde intermediate in recently characterized BMCs: propionaldehyde in the fucosome [24] and lactaldehyde in the PVM BMC [25].

Recently, comparison of experimentally characterized catabolic BMCs led to the identification of their common core biochemistry (Fig. 1B) [25]. The metabolic function of the BMC (e.g. propanediol utilization) is defined by the aldehyde-generating enzyme (e.g. propanediol dehydratase) which we refer to as the “signature” enzyme (Fig. 1). The aldehyde is acted on by an NAD^+^ and CoA-dependent aldehyde dehydrogenase (AldDH), forming NADH and an acyl∼CoA product [29], [30]. Because this reaction is encapsulated, the cofactors must be recycled within the metabolosome [31], [32]. In order to regenerate NAD^+^, an alcohol dehydrogenase (AlcDH) reduces a second aldehyde, forming an alcohol [28], [31]. A phosphotransacetylase (PTAC) then acts on the acyl∼CoA to replace the CoA moiety with a phosphate [32], [33], which can then participate in substrate-level phosphorylation. Because the biochemical core is a requirement for cofactor recycling, we hypothesize that all BMCs that utilize an acylating AldDH also require the other core enzymes, and we refer to the BMCs that encapsulate these core biochemical transformations as metabolosomes. Accordingly, two functional paradigms for BMCs have emerged: anabolic carboxysomes, which encapsulate RuBisCO and carbonic anhydrase, and catabolic metabolosomes, which encapsulate the aforementioned core enzymes for the specific purpose of recycling cofactors (Fig. 1).

In experimentally characterized alpha-carboxysome and metabolosome loci, the essential BMC components (shell proteins, signature and core enzymes; Fig. 1) are encoded within a genetic locus. This has implications for horizontal gene transfer. The PDU locus has been suggested to instantiate the concept of a selfish operon: a group of contiguous genes that functions together [34]. Such a self-sufficient genetic and metabolic unit is likely to confer a beneficial skillset to a host organism in a single horizontal gene transfer event [34]. Substantiating this hypothesis, it has been shown that the PDU locus alone is necessary and sufficient to form a fully functional organelle that provides the host organism with new metabolic potential [35]. This attribute of BMC loci is also significant for synthetic biology; engineered BMC loci can be envisioned as both genetic and metabolic modules for plug and play introduction of biochemical pathways of industrial interest [36].

The increasing availability of genomic sequence data enabled the discovery that many bacteria potentially form organelles. The identification of BMC shell protein genes, gene cluster conservation and genomic context were used in the first surveys of putative BMC functions and their distribution [3], [37]. As the number of available microbial genomes increased, a correlation network for co-occurrence of protein functional groups within BMC loci was devised [38]. A major limitation of this method was its gene-centric approach; loci could only be predicted manually after visualizing the resulting co-occurrence network of genes. Although this analysis effectively recovered a number of loci that had been experimentally characterized, the reliance of the method on co-occurrence of protein functional groups made it insensitive to rare BMC locus types. As a result, several BMC types that had been previously described based on genomic context-based approaches [3], [37] were missed. Furthermore, the presence of highly abundant functional groups in multiple different BMC locus types (such as alcohol dehydrogenases) posed a problem because the clustering step sought to assign these functional groups to a single BMC locus type. This resulted in co-occurrence networks that do not represent the true co-occurrence of genes within a given locus type and underestimation of BMC diversity. The most recent gene-centric bioinformatic survey of BMC loci divided them into different groups based on pfams of known signature enzymes but did not divide them into functional sub-types, predict new signature enzymes, or define novel BMC locus types [39].

Here we present the results of a new approach, LoClass (Locus Classifier), to surveying and classifying BMC loci with an emphasis on discovery of novel BMC locus types and variants of the paradigmatic types. It is a novel locus-centric method for predicting and sorting loci through the generation of a locus similarity network. To compare loci, we approximate the functional attributes of a locus by representing it as the set of pfam domains encoded by genes in the locus. While the use of pfams to visualize the functionality in a locus is relatively low-resolution, in that one pfam may correspond to a variety of homologous sequences of different functions, this coarse resolution allows for the recognition of pfams corresponding to the functionally similar biochemical cores. Moreover, the lost resolution is regained through the multiplicity of pfams conferring organelle-supporting functions present in any given locus; the diversity among these pfams aids in distinguishing different BMC locus types and sub-types. By focusing on the regions flanking BMC shell protein genes instead of being confined to presumed operons, we are able to circumvent the lack of transcriptional data for the majority of sequenced genomes that contain BMCs. LoClass also captures the genomic neighborhood of BMC shell protein genes, revealing that genes encoding the organelles are frequently situated in the context of other genes that provide ancillary functions, such as regulation, co-factor synthesis, or transport for BMC substrates. Recognition of these gene products and their roles in supporting BMC function will be useful both in functionally characterizing diverse BMCs and in the design of BMC locus modules that are ready for “plug and play” applications. Finally, LoClass is able to perform direct comparisons and classification of loci, granting high sensitivity to rare locus types. The result is a comprehensive taxonomy of BMC locus (sub)types and the identification of several novel putative BMC locus types, including one that we predict extends BMC functions beyond the paradigmatic metabolosomes and carboxysomes.

## Materials and Methods

### Predicting BMC Shell Protein Genes

The standard PF00936 hidden markov model (HMM) is often not robust for the identification of N-terminal cryptic BMC domains of CsoS1D/CcmP-type BMC-T proteins, which have only weak sequence similarity to other BMC domains [10], [13]. Accordingly, a modified PF00936 HMM was built using the seed alignment (128 sequences) in the Pfam database [12] with five cryptic BMC-T domain sequences (NCBI protein accessions: YP_007073159.1, YP_007144647.1, YP_473976.1, YP_637369.1, YP_925211.1) added to the alignment (a total of 133 sequences) to render it more robust for detecting these cryptic BMC domains (Dataset S3, Dataset S4). This expanded PF00936 HMM and the PF03319 HMM were searched using *hmmsearch* in the *hmmer* [40] package against a local copy of the NCBI Non-redundant Protein Database (NR) downloaded from NCBI on July 3rd, 2013. All hits with an e-value less than or equal to 1e-05 that corresponded to genomic records from the Genbank, RefSeq, EMBL, and DDBJ databases were accepted as BMC shell proteins homologs. This cutoff was chosen based on manual inspection of results for spurious hits relative to various e-value thresholds. Identical proteins to each of these hits in the genomes containing these hits were retrieved from NCBI using NCBI Entrez Programming Utilities [41] because NR frequently stores identical proteins in a single record. Where at least one BMC protein in a given genome was non-identical to all other proteins included in this analysis, that genome was included, and this step retrieved all other BMC proteins in its genome that might be identical to other proteins included in this analysis. Where every BMC protein in a given genome was 100% identical to a protein already included in this analysis, the BMC proteins from this genome were not included. This reduced the computational and analytical burden introduced by the analysis of likely identical loci from very closely related genomes. The genome of the model cyanobacterium *Synechococcus elongatus* PCC 7942 (Syn7942) was not included in this analysis due to the formatting irregularities of its sequence records. The closely related organism *Synechococcus elongatus* PCC 6301 (Syn6301), whose BMC proteins are identical to those of Syn7942, was included. We examined the similarity of these genomes using NUCmer in the MUMmer 3.1 [42] package. These genomes are highly syntenic, the greatest differences being a 188.9 kb inversion in the genome sequence and a 231 bp sequence in Syn6301 that is absent from Syn7942, confirming a previous genomic comparison [43]. Sequence identity between the two genomes over all aligned regions was 99.9%. Syn6301 was therefore used as a proxy for Syn7942.

### Generating Prospective BMC Loci

**Figure 2:**
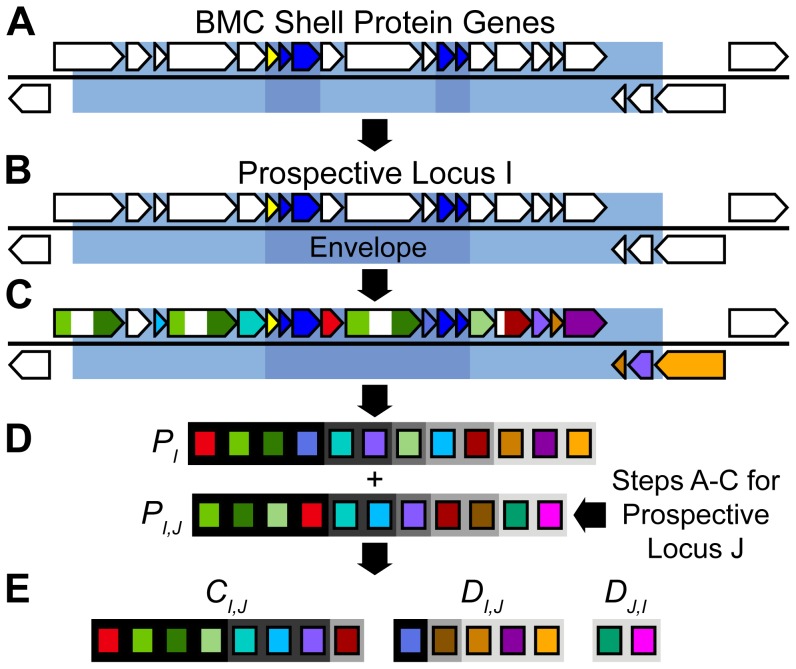
Simplified workflow of LoClass for locus similarity network generation. (A) After genes encoding BMC shell proteins (PF00936, dark blue; PF03319, yellow) are identified using *hmmsearch*, their position on the chromosome is determined. The region 10 kb upstream and downstream of each PF00936 and PF03319 domain is considered a Prospective BMC Locus (pale blue). The envelope (blue) is defined as the maximal portion of the Prospective BMC Locus bounded by BMC shell protein genes. (B) Where Prospective BMC Loci overlap, they are merged into one Prospective Locus. (C) All non-shell protein genes in the Prospective Locus are searched against Pfam [12]. Pfam hits are represented by colored regions of the genes. Genes without pfams hits (white) are not considered. (D) Loci are represented by their pfam set, excluding genes containing PF00936 and PF03319 domains. Pfams, represented by colored rectangles, are weighted based on their relative distance from the envelope. This distance weight is represented by the darkness of the background behind the rectangles, where a black background corresponds to a pfam found inside the envelope with a weight of 1, and where a light grey background corresponds to a pfam separated from the envelope by at least four open reading frames with a weight of 0.6. *P_I_* is the set of pfams found in Locus I, while *P_J_* represents the set of pfams found in a different Locus J (not shown). (E) By comparing the sets of pfams *P_I_* and *P_J_*, we determine the set *C_I,J_* of common pfams to both loci and the two sets *D_I,J_* and *D_J,I_* of pfams unique to Locus I and Locus J, respectively. These three sets, along with the distance weight and the other weights (Materials and Methods) are then used to calculate the locus similarity score between these two loci.

Prospective BMC Loci were initially defined as the region on the chromosome 5 kb upstream and downstream of each BMC shell protein gene, analogous to the five open reading frame distance used by Jorda et al [38] to define BMC-related gene co-occurrence. However, the conserved locus of several characterized BMC locus types (e.g. the alpha-carboxysome locus of *Halothiobacillus neapolitanus*) contain a gap greater than 10 kb between BMC-related genes and the nearest BMC shell protein gene. This resulted in several characterized loci being incomplete. Prospective BMC Loci were then redefined as the region 10 kb upstream and downstream of BMC shell protein genes (Fig. 2A). Wherever these BMC loci overlapped with each other, they were merged into one Prospective BMC Locus (Fig. 2B). We defined the envelope as the largest region in the locus flanked by BMC shell protein genes (Fig. 2B). All genes that at least partially overlapped a given locus range were deemed part of the locus. All loci that were truncated by the beginning or end of the scaffold were designated as potentially incomplete loci and were not included in subsequent analyses.

### Identifying Pfams in Prospective BMC Loci

A local copy of the Pfam 27.0 database [12] was searched against the proteins corresponding to all non-shell protein genes found in a given BMC locus using *hmmsearch* with a loose e-value cutoff of 0.01 (Fig. 2C). Where a pfam alignment in a protein sequence overlapped a pfam alignment in the same sequence by over 50% of the pfam length, the alignment with the higher e-value was discarded. The pfam sets found in each locus were then used to compare it to each other locus using a novel scoring mechanism (Fig. 2D).

### Building the Locus Similarity Network

A novel scoring method we name LoClass, the Locus Classifier, was developed to determine the relative similarity of each locus to every other locus. The scoring mechanism used has a positive component, determined by the pfam domains any two loci share in common, and a negative component, determined by the pfam domains present in only one of the two loci. Let *L* represent the set of all BMC loci, where *P_I_* is the set of all pfams found in the locus *I* (Fig. 2D) and *p* represents a given pfam domain. The set of pfams common to loci *I* and *J* can then be represented as
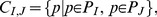
(1)while the set of pfams found in *I* but not *J* can be represented as
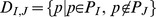
(2)(Fig. 2E). Various weights comprise the positive and negative scoring components. First, since the shared pfams between two loci are more important for determining their similarity than their disjoint pfams, a weight *k* is applied to the negative score. Testing various values for *k* showed that for these loci, a weight of 0.5 yielded the best results, as judged by the recapitulation of the experimentally confirmed PDU, EUT, and PVM locus types.

If *d* represents the minimum number of open reading frames between the envelope and a gene that contains the pfam, then a distance weight can be applied to account for the decreasing likelihood that genes in a locus are related to the function of the BMC the further they are from the envelope (Fig. 2D). This distance weight can be represented by
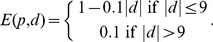
(3)Some pfams may be used to identify BMC loci described in the literature: PF12288 (CsoS2) and PF08936 (CsoSCA) are found specifically in alpha-carboxysomal loci [44], and PF00132 (CcmM) and PF00132 (CcmN) are found specifically in beta-carboxysomal loci [45]. Likewise, PF06751 and PF05985 (ethanolamine ammonia lyase subunits) comprise the signature enzyme ethanolamine ammonia lyase for the ethanolamine utilization locus, while PF02286, PF02287, and PF02288 (propanediol dehydratase subunits) identify the signature enzyme for the propanediol utilization locus. PF00596 corresponds to an aldolase predicted to be the signature enzyme for the Planctomycetes and Verrucomicrobia microcompartment (PVM) locus type [25]. This prior knowledge is incorporated into the score by designating these pfams as identifying pfams and creating an identifying pfam weight
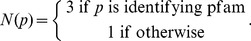
(4)Some pfams, such as PF00171 representing aldehyde dehydrogenase, PF06130 representing PduL phosphotransacetylase [46], and PF00465 representing iron-containing alcohol dehydrogenase are extremely common among BMCs and therefore do not reveal much about the similarity of two loci. Likewise, if two loci both contain a pfam that is found in very few BMC loci, this more strongly indicates that these two loci are similar, while if only one contains the rare pfam, this may indicate that they are quite different types. We therefore add a rare pfam weight
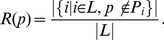
(5)Since *R(p)* represents the proportion of BMC loci that do not contain the pfam *p*, the weight approaches but never reaches 1 for very rare pfams and 0 for very common pfams.

Finally, when a pfam is only found in one of the loci, it is necessary to consider whether that pfam is frequently found within loci that also contain the set of pfams that the two loci share in common. This aids in down-weighting secondary pfams that are not necessary for the core function of a BMC, are not always localized in the BMC loci of a given type, or are only occasionally found near the periphery of a BMC locus while not actually being related to the BMC function. This co-occurring pfam weight can be represented by
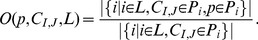
(6)Applying the appropriate weights to the positive and negative score components, we define a similarity score between any two loci as
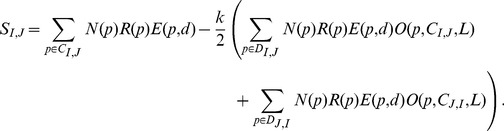
(7)This score was generated for each locus pairwise comparison in this analysis, and these scores were then used to construct the edge lengths for a BMC locus similarity network.

### Identification and Classification of Satellite and Satellite-Like BMC Loci

Analysis and clustering of the locus similarity network was complicated by the presence of what we term satellite loci, loci that encode an insufficient complement of BMC shell protein genes (only encoding either PF03319 or PF00936 domains, but not both) to form a BMC; genomes containing satellite loci putatively encode additional shell proteins and possibly other BMC components for a Candidate/Confirmed BMC in another locus. We define satellite BMC loci as those that meet three conditions: the locus contains an insufficient shell protein gene complement, all the shell protein genes in the locus are contiguous to each other, and there is at least one other non-satellite BMC locus in the genome. In order to prevent false positives, predicted satellite loci were manually inspected for the presence of genes with common BMC-associated pfams immediately nearby the BMC shell protein genes. Where such genes were found or where the locus met all requirements except that there was more than one locus in the genome, these loci were labeled as satellite-like loci. Where a signature enzyme was present or a putative function had already been assigned to the locus in the literature, as with the putative ethanol utilization locus, type names were assigned to these loci (see below) [47]. To simplify the analysis of the locus similarity network, predicted satellite loci were excluded from further steps of the analysis, while satellite-like loci, which we predict encode a structurally incomplete BMC locus, were included. This greatly reduced the spurious results in the clustering step.

### Clustering BMC Loci

The locus similarity network of BMC loci was visualized in Cytoscape 2.8.3 [48] at a score cut-off of −20 using the Force-directed layout, where the edge lengths corresponded to the locus similarity score determined above and normalized within Cytoscape. The resulting network was then clustered using multiple iterations of the Markov Cluster Algorithm (MCL) [49] at increasing stringency using the *clusterMaker* [50] plug-in in Cytoscape. MCL has been used successfully in clustering various types of biological networks, such as sequence similarity, protein expression profiles, and scientific article similarity [51] and is best applied to undirected networks where edges represent similarity [49]. Stringency in MCL is controlled by manipulating the score cut-off and the inflation value, where the score represents the metric used to calculate edge length and the inflation value is a property of MCL; a higher inflation value will effect a more fine-grained clustering [49].

**Figure 3:**
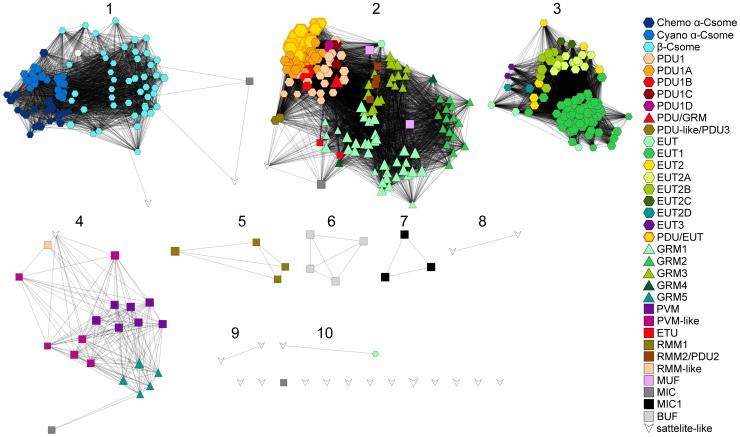
Similarity network of bacterial microcompartment loci. Nodes represent all Candidate BMC Loci and satellite-like loci analyzed using LoClass. The length of any given edge between two nodes is proportional to the pairwise locus similarity score as generated using the LoClass method. The locus similarity network was clustered using MCL at a score cut-off of 3 and inflation value of 2, resulting in 10 different clusters. Node sizes are proportional to the number of genes in the envelope, the maximal region in the locus bounded by BMC shell protein genes. Node colors and shapes correspond to the locus (sub)type as predicted by our analysis (see key). The white circle in Cluster 1 indicates a locus in a synthetic genome not included in our analysis [121].

Resulting clusters were then assigned numbers (Fig. 3). Whenever a cluster still contained obvious sub-groups, another iteration of MCL sub-clustering was performed at more stringent cut-offs until the resulting clusters were either too small to be informative, representing nearly identical loci, or until the cluster was so tight that the score cut-off necessary to split up the cluster exceeded 40. This process of varying the inflation value and score cut-off to achieve relevant clusterings has been well described [51]. At each level of sub-clustering, an additional numeral was added to the cluster number. For example, the first cluster resulting from the sub-clustering of Cluster 1 was called Cluster 1.1. The cut-offs used and resulting clusters are shown in Figure S1 and its legend, and the resulting network is included in Dataset S5.

### Assigning Type Names to BMC Loci

At each level of clustering, the loci were manually compared with previously studied or predicted BMC loci. Where all of the loci in a large cluster appeared to be of the same type as a previously studied locus (e.g. EUT), those loci were first assigned that type name as a super-type. Where that cluster was further sub-clustered once or twice, the loci in those clusters were additionally assigned a numeral followed by an alphabetic character (e.g. EUT1, EUT1A). In our taxonomy, all loci with a different numeral are deemed different types, while those whose type names are differentiated only by the alphabetic character (e.g. EUT2A, EUT2B) are deemed different sub-types. Where a locus (sub)type clustered with loci of a known type but either was lacking the signature enzyme in the pathway or bore significant differences from the type, that locus was designated to be “like” that BMC type (e.g. PDU-like). Where a locus type had not previously been named in the literature, if all genomes containing that type came from a distinct set of taxa that do not contain any other types, we assigned that type a three-letter name based on those taxa (e.g. RMM1). Where multiple loci of unknown function did not meet any of these prior criteria, they were designated Metabolosome of Unknown Function (MUF), Metabolosome with an Incomplete Core (MIC), or BMC of Unknown Function (BUF) type, depending on whether they contained all core metabolosome genes, only the AldDH but not a complete core, or none of the metabolosome core enzymes, respectively. Where multiple MIC loci clustered together, an additional numeral was assigned to the name to designate that specific type (MIC1).

### Summarizing Pfam Occurrence and Selecting Representative Loci

**Figure 4:**
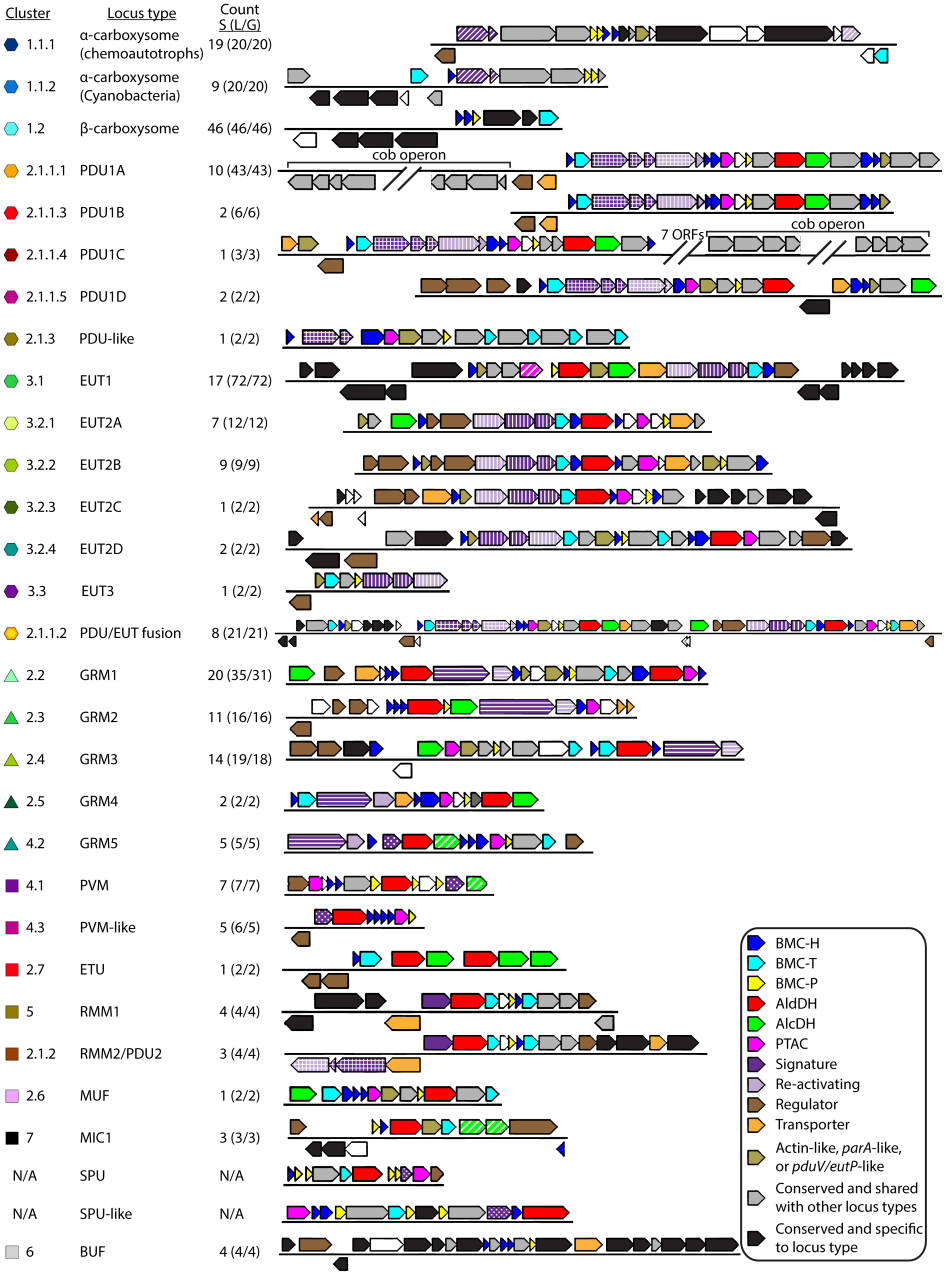
Representative BMC Loci. Cartoon representation of the most highly conserved contiguous region of the Representative Loci, in order of appearance in the text. Where a (sub)type is dominated by many highly syntenic examples from one or two species, locus bounds were chosen based on conservation across all species in the (sub)type. Locus statistics are represented in the “S (L/G)” column: “S” represents number of species that contain the locus, “L” represents the number of loci, and “G” represents the number of genomes that encode the locus. Genes are color-coded according to their annotation: blue, BMC-H; cyan, BMC-T; yellow, BMC-P; red, aldehyde dehydrogenase; green, iron-containing alcohol dehydrogenase; green diagonal hash, other putative alcohol dehydrogenases; solid pink, *pduL*-type phosphotransacylase; pink diagonal hash, *pta*-type phosphotransacylase; purple diagonal hash, RuBisCO large and small subunits; purple vertical hash, ethanolamine ammonia lyase subunits; purple crosshatch, propanediol dehydratase subunits; purple horizontal hash, glycyl radical enzyme and activase; dotted purple, aldolase; solid purple, aminotransferase; brown, regulatory element including two-component signaling elements; orange, transporter; teal, actin/*parA*/*pduV*/*eutP*-like. Genes colored gray indicate that the gene is present in over 50% of members in the locus (sub)type described (e.g. GRM1), and are in over 50% of members of at least one other locus (sub)type (e.g. found in GRM1 and GRM3). Genes colored black indicate that the gene is present in over 50% of members in the locus (sub)type described and not present in over 50% of members of any other locus (sub)type. Genes colored white are those that are present in the Representative Locus but are not present in over 50% of members of that locus (sub)type. Representative Loci are highlighted in yellow in Dataset S1.

For each locus (sub)type, a representative locus that best captures the pfam diversity of all loci of that (sub)type was chosen (Fig. 4; Dataset S1). For each pfam that appears in any of the loci of a given (sub)type, the proportion of loci of that (sub)type that contain that pfam was calculated (Fig. S2). Then, for each locus, a representative score was calculated by adding these proportions for all pfams it contains and subtracting the proportions for all pfams it lacks. The locus with the highest score was chosen as the representative locus for that (sub)type; where multiple loci corresponded to the highest representative score, a characterized BMC locus or a BMC locus from a reference genome, in that order of preference, was selected as the representative. If one or more pfams present in the majority of the loci were not encoded in the highest scoring locus but were encoded in the next highest scoring locus, then the second locus was selected as the representative. These loci generally exemplify the consistent trends across a locus (sub)type, as well as the unique trends present in a subset of the loci. However, where there is a great deal of diversity within a given locus (sub)type, as is the case with alpha- and beta-carboxysomes, many loci of that (sub)type may differ substantially from the representative locus.

### Analysis of Loci from Candidate Phyla

In order to identify BMC loci in genomes from candidate phyla not archived in NR, we examined the single-cell genomes from the recent GEBA-MDM project [52] for the presence of BMC shell protein genes using the Integrated Microbial Genomes (IMG) Database [53]. In addition, we searched for the presence of BMCs in all unclassified bacterial phyla in IMG. These loci were then manually inspected and compared to the BMC locus taxonomy constructed by LoClass from data in NR.

### Aldehyde Dehydrogenase Phylogenetic Tree

**Figure 5:**
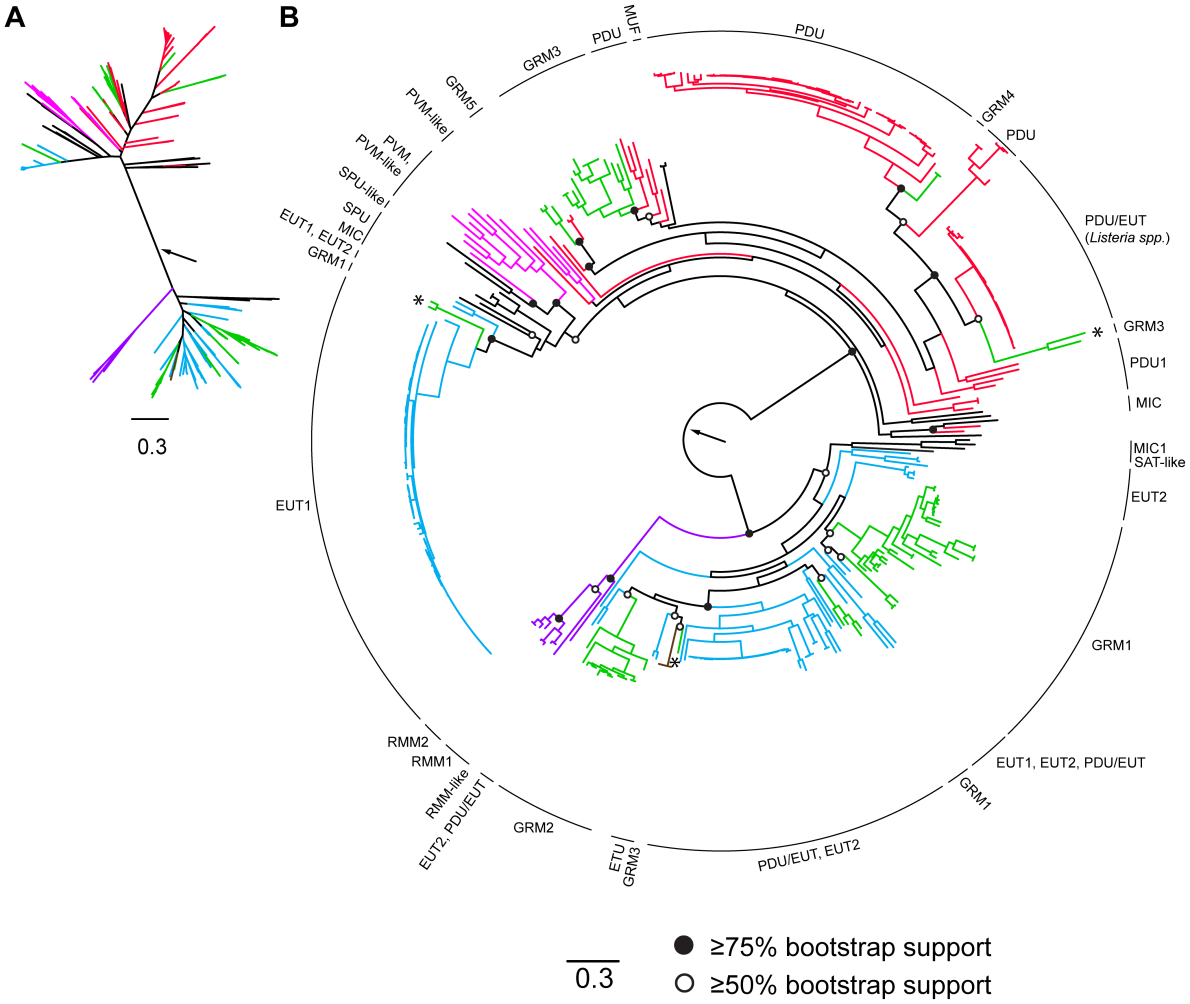
Phylogeny of aldehyde dehydrogenases. Tree root is denoted by arrow. Branches are color coded according to general classification of the locus in which the aldehyde dehydrogenase is encoded: red, PDU; cyan, EUT; green, GRM; pink, PVM and PVM-like; purple, RMM; brown, ETU; black, MUF and others. SAT-like refers to satellite-like loci. Asterisks (*) annotate outlier taxa discussed in text. If bootstrap support of a node separating branches of differing color were above 50% or 75%, they were denoted by open or closed circles, respectively. Scale bar represents number of substitutions per position.

Sequences for aldehyde dehydrogenase genes within BMC loci (accession numbers in Dataset S1) were aligned using MUSCLE [54], [55]. The alignment was visualized and edited using Jalview [56]. All sequences that did not contain the catalytic cysteine (see Results and Discussion) were removed from the alignment. In addition, sequences from *Shigella sonnei* (NCBI protein accessions: YP005456972.1, YP310962.1) and *Roseburia inulinivorans* (EEG94445.1) were significantly shorter than the rest of the sequences and were also removed (Dataset S6). The alignment was manually curated by removing all gapped positions. A maximum likelihood phylogenetic tree was constructed by using PhyML [57] in the phylogeny.fr web server [58], [59] with 100 bootstraps (Fig. 5). Four sequences (IMG Gene OID: 2264936618; NCBI protein accessions: YP_004405262.1, YP_007299416.1, CCK75300.1) were significantly divergent, forming extremely long branches, and were removed from the alignment for the final tree.

### BMC Phylum Tree

Genome assemblies of 2,025 bacteria were scanned for homologs of a set of 38 universally conserved single-copy proteins present in Bacteria and Archaea [52]. The assemblies were translated into all six reading frames, and marker genes were detected and aligned with *hmmsearch* and *hmmalign* included in the HMMER3 [60] package using HMM profiles obtained from PhyloSift [61]. Extracted marker protein sequences were used to build concatenated alignments of up 38 markers per genome.

**Figure 6:**
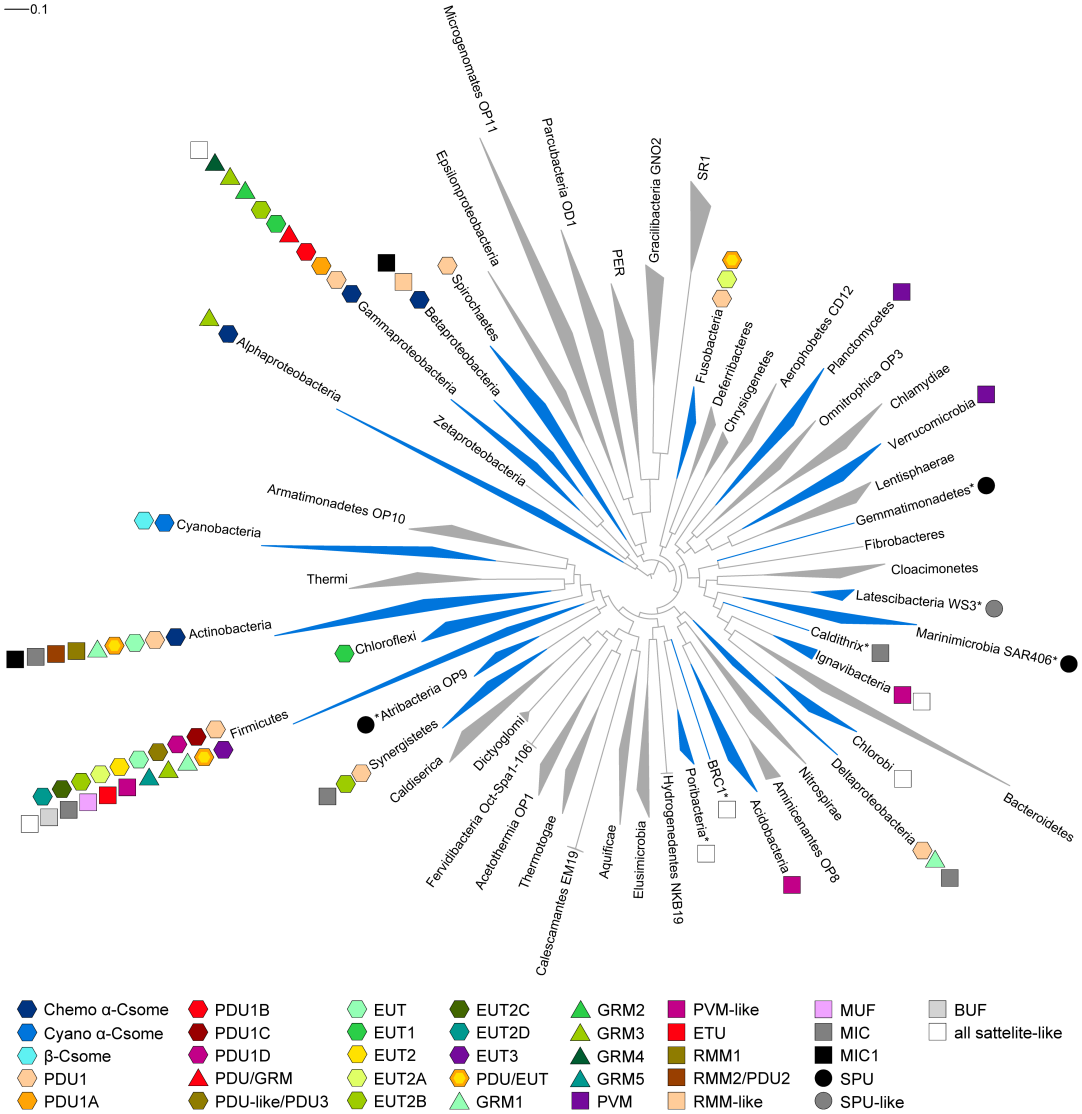
Bacterial phyla tree with distribution of BMC locus types. The classified BMC locus types, excluding satellite and satellite-like loci, denoted as colored shapes are adjacent to the phyla in which they appear. For a given phylum, the shape of the triangular wedge represents sequence diversity; the nearest edge represents the shortest branch length from the phylum node to a leaf, while the farthest edge represents the longest branch length from the phylum node to a leaf. Phyla marked with an asterisk (*) are not in NR but contain BMC loci; the data were retrieved from IMG (Materials and Methods). Phylum tree based on [52] with expansion by Christian Rinke.

The phylogenetic inference method used was the maximum likelihood based FastTree2 [62] with CAT approximation with 20 rate categories and Jones-Taylor-Thorton (JJT) for FastTree2. Sequences were grouped into clades and manually corrected in ARB [63]; newick trees were exported from ARB and beautified with iTOL [64]. BMC locus (sub)types were then mapped onto the resulting phylum tree (Fig. 6).

## Results and Discussion

### Summary of BMC-Containing Loci and Description of Nomenclature

The numbers of genomes and loci analyzed using LoClass, as well as definitions of terms used in this Discussion are given in Table 1. BMC loci are prevalent among Bacteria, found in a total of 23 bacterial phyla (Fig. 6). Sixteen phyla were identified by searching against NR and analyzing using LoClass; seven additional phyla were found after inclusion of BMC loci identified in candidate phyla and single-cell genomes from IMG (Fig. 6). The greatest diversity of BMC locus (sub)types is in the Firmicutes and Gammaproteobacteria; however, these phyla also contain the highest numbers of BMC-containing genomes. Many of these (sub)types also appear in distantly related phyla (Fig. 6, Fig. S3). This distribution is consistent with the hypothesis that BMC loci are frequently horizontally transferred.

**Table 1:** Definitions of terms and counts for locus and genome categories analyzed using LoClass.

Term	Definition	Count
Locus Super-type	The largest cluster of loci where all contained loci encode the same signature enzyme or are syntenic to these loci while encoding no alternative signature enzyme.	15
Locus Type	A more stringent cluster of loci; in addition to conserved signature enzymes, these loci typically are syntenic to one another, indicating common origin.	23
Locus (Sub)type	The most stringent clustering of loci, where many syntenic loci of the same type are separated into smaller groups; these typically vary by genes encoding ancillary functions.	30
Prospective BMC Locus	Region of a genome including BMC shell protein genes within 20 kb of each other and all other genes within 10 kb upstream and downstream.	580
Envelope	Region of a Prospective BMC Locus that includes only the BMC shell protein genes and genes encoded between them.	-
Satellite loci	Prospective BMC Loci that are predicted to encode a subset of shell components for a BMC and no other BMC-related genes (Materials and Methods).	149
Satellite-like loci	Prospective BMC Loci that meet most but not all of the criteria established for satellite loci or encode other BMC-related genes.	21
Confirmed BMC Loci	All Prospective BMC Loci which are of the same Locus Type as a locus whose function has been experimentally elucidated.	335
Candidate BMC Loci	All Prospective BMC Loci that are not putative satellite, satellite-like, or Confirmed BMC Loci.	75
Carboxysome loci	Confirmed Loci encoding the carboxysome.	87
Metabolosome loci	Candidate/Confirmed Loci that, of the core metabolosome enzymes, encode at least a core AldDH or are members of a Locus Type where the majority of loci do. These loci presumably encapsulate catabolic reactions (Fig. 1B).	312
Metabolosome loci with an incomplete core	Metabolosome loci that are of a Locus Type where the majority of loci do not encode a core AlcDH and/or PTAC.	22
BMC-containing genomes	Genomes that contain any Prospective BMC Loci.	329
Satellite-containing genomes	Genomes containing at least one putative satellite locus.	77

Counts for Locus super-types, types, and sub-types include loci from IMG that were analyzed manually. Due to the fragmentation of most IMG loci, these were excluded from all other counts.

Six genomes contain the highest number (five) of total BMC loci in one genome (Table S2). Subtracting the satellite and satellite-like loci in these genomes reduces this number to two or fewer Candidate/Confirmed Loci. Among all genomes surveyed, as many as three functionally distinct Candidate/Confirmed BMC Loci are found within a single genome; this occurs in 12 genomes surveyed (Table S2). Nearly every outlier in our analysis, including known and predicted cyanobacterial satellite BMC loci [16], fulfill all three of the criteria for satellite loci (see Materials and Methods; Dataset S1).

### Shell Protein Composition of Loci

Based on the experimental data [65], [66] and structural models of BMCs, the number of BMC-H (a major component of the shell) genes is expected to be larger than that for BMC-T (presumably minor shell components) and BMC-P (only required to cap the vertices). We observed that each genome contains 3.5 BMC-H genes, 1.4 BMC-T genes, and 1.2 BMC-P genes on average per Candidate/Confirmed BMC Locus. The largest number of BMC-H genes predicted in any one genome (fifteen) is in *Clostridium saccharolyticum* WM1 (Table S2), distributed across three Candidate/Confirmed Loci. Four genomes contain the highest count (five) BMC-T genes in any genome: *Desulfosporosinus orientis* DSM 765, *Desulfosporosinus meridiei* DSM 13257, and two strains of *Clostridium kluyveri* (Table S2); these are distributed across two to three Candidate/Confirmed BMC Loci. *Melioribacter roseus* P3M-2 exceeds every other genome in BMC-P count, with a total of seven genes spread out across five loci, four of which are satellite/satellite-like loci. No other genome contains more than three BMC-P genes. While we found that BMC-H genes typically outnumber BMC-T and BMC-P genes, this is not always the case. The most extreme example is *Haliangium ochraceum* DSM 14365, which contains three BMC-T, three BMC-P genes, and only one BMC-H. Despite the unusual proportion of BMC shell protein gene types in this genome, recent expression of the seven *H. ochraceum* shell proteins in *E. coli* resulted in remarkably homogeneous and stable BMC shells that could be readily purified in large quantities [67].

### The Alpha- and Beta-Carboxysome Loci (Cluster 1)

The two types of carboxysomes are named for the form of encapsulated RuBisCO; alpha-carboxysomes encapsulate form 1A RuBisCO, beta-carboxysomes encapsulate form 1B RuBisCO [45]. Alpha-carboxysome loci are found in the phylum Cyanobacteria, as well as in some chemoautotrophs from the phyla Actinobacteria, Alphaproteobacteria, Betaproteobacteria, and Gammaproteobacteria (Fig. 6, Table S1). The common core of all alpha-carboxysome loci consists of genes for the RuBisCO large (CbbL) and small (CbbS) subunits, a beta carbonic anhydrase CsoSCA [68], a protein of unknown function CsoS2, and an accessory protein, a pterin dehydratase-like RuBisCO assembly factor [69] (Fig. 4, Dataset S1).

The alpha-carboxyome loci of chemoautotrophs and cyanobacteria are distinguished by differences in the genes flanking the conserved core (Fig. 4). For example, most chemoautotrophic loci encode a protein 27–39% identical to the LysR family regulator CbbR (UniProtKB: P52690) [70], while no cyanobacterial alpha-carboxysome loci do, presumably because the carboxysome is constitutively expressed. This gene is usually encoded immediately upstream of *cbbL* and, in two of these loci, has been shown experimentally to regulate and be divergently transcribed from the carboxysome operon [71], [72] (Fig. 4, Dataset S1). Other proteins encoded in over half of the 20 chemoautotroph carboxysome loci are bacterioferritin, the accessory proteins CbbO and CbbQ involved in RuBisCO activation, a UPF0753 family protein of unknown function, and a homolog to the chromosome partitioning protein ParA (Dataset S1). ParA has been implicated in spatial arrangement of beta-carboxysomes in the cyanobacterium *Synechococcus elongatus* PCC 7942 [73]. Approximately half of the loci in chemoautotrophs encode a protein 34–39% identical to NdhF (UniProtKB: P31971), part of the NDH-1 complex; some paralogs of NdhF are involved in CO_2_ uptake (reviewed in [74]; [75]).

Cyanobacterial alpha-carboxysome loci are found in the *Prochlorococcus* and marine *Synechococcus* genomes (Table S2). In addition to the core alpha-carboxysome proteins, these loci always encode a Ham1 family protein of unknown function on the opposite strand. (Dataset S1). Furthermore, nearly all of these loci encode the light-independent (dark-operative) protochlorophyllide reductase (DPOR) subunits ChlB, ChlN, and ChlL and the light-dependent protochlorophyllide reductase (LPOR) (Dataset S1). However, the benefit of co-localizing the LPOR and DPOR chlorophyll biosynthesis systems with the carboxysome operon is unclear. The NDH-1_3_/NDH-1_4_ CO_2_ uptake system, which includes NdhD, NdhF, and ChpX/Y (reviewed in [74]), is encoded in the marine *Synechococcus* loci (Dataset S1).

Beta-carboxysome loci vary significantly from one another, as depicted by the loose locus similarity network (Fig. S1). In addition to the shell proteins CcmK and CcmL, the only proteins encoded in every beta-carboxysome locus are the γ-carbonic anhydrase homolog, CcmM [76], and CcmN [37] (Fig. 4, Dataset S1); both are essential for beta-carboxysome formation [17], [37], [77]. Interestingly, the carboxysome signature genes, the RuBisCO large and small subunits (RbcL and RbcS), are only encoded in approximately a quarter of beta-carboxysome loci (Fig. S2), almost always with the RuBisCO chaperone RbcX (Dataset S1). In contrast, the presumed accessory genes for inorganic carbon uptake, the NDH-1_3_/NDH-1_4_ gene cluster present in the marine *Synechococcus* alpha-carboxysome loci, are encoded in 70% of the beta-carboxysome loci (Fig. 4, Dataset S1).

### The BMC Loci of Heterotrophs

Certain pfams are highly abundant across many different heterotrophic BMC loci. Notably, the majority of Candidate/Confirmed BMC Loci analyzed with LoClass (excluding carboxysome loci) contain the metabolosome core genes (Fig. 1B) for AldDH (PF00171; 94%), AlcDH (PF00465; 76%), and PduL (PF06130; 66%), a PTAC, as well as PduV/EutP proteins of unknown function (PF10662; 68%). 57% of satellite-like loci encode a PduV/EutP pfam nearby the BMC shell protein gene(s) (Dataset S1).

### The Propanediol Utilizing (PDU) Loci (Cluster 2.1)

The PDU1A-D loci are relatively syntenic (Fig. 4); the sub-types generally group phylogenetically but are confined to two phyla (Fig. 6, Table S1). PDU1A loci include the experimentally characterized propanediol utilization operon found in *Salmonella enterica* [22] (Table S2). The PDU1A and C loci contain part of the *cob* operon, which encodes the accessory function of synthesizing cobalamin (Dataset S1). Cobalamin, or vitamin B_12_, is a required cofactor for propanediol dehydratase, the signature enzyme of the PDU loci [78].

Other PDU1 sub-types are typically distinguished by the absence of PDU1A ancillary genes. For example, PduS oxidoreductase, which is involved in cobalamin biosynthesis [79], is not found in PDU1C loci. PduW, a propionate kinase, is absent from PDU1B loci (Fig. S2). The PocR regulatory protein is not present in PDU1C and PDU1D sub-types. An interesting difference between PDU1D loci and other PDU1 loci is the presence of a putative two component regulatory system, in which the histidine kinase contains a PocR-domain (Dataset S1). The PocR domain is also found in the AraC family regulatory protein of the PDU1A loci as well as in several other uncharacterized regulatory proteins [80]. This is a previously undescribed regulatory mechanism for the PDU metabolosome, though a similar PocR-domain containing two-component system has been described for the 1,3-propanediol synthesis operon in *Clostridium butyricum* [81].

Cluster 2.1 also contains a PDU-like locus, found in *Clostridium kluyveri*. This appears to be a reduced PDU locus, lacking the medium subunit (PduD) of the diol dehydratase, and the core AldDH and AlcDH enzymes (Fig. 4, Fig. S2). Another unique feature is the presence of four PduS homologs (Fig. 4). The additional AlcDH and AldDH necessary to form a metabolosome core enzyme set may be supplied by the ethanol utilizing (ETU) locus (Fig. 4; discussed below) elsewhere in the genome. Additional observations and comparisons of the various PDU locus (sub)types are included in Text S1.

### The Ethanolamine Utilizing (EUT) BMC Loci (Cluster 3)

EUT1 loci contain the experimentally characterized ethanolamine utilization operon in *Salmonella enterica*. All EUT1 loci except for one are found in the Gammaproteobacteria (Table S1, Table S2), the majority in pathogenic enterobacteria. The EUT2 loci form a much looser similarity network than those of EUT1 (Fig. S1), indicating a great deal more variety between the loci. While nearly all EUT1 loci are found in one phylum, EUT2 sub-types are found in organisms inhabiting diverse environments; representatives are found in four phyla, the majority in Firmicutes (Table S1, Fig. S3). EUT3 loci are found only in two *Desulfitobacterium hafniense* strains. All three of the EUT locus types encode both subunits of the signature enzyme ethanolamine ammonia lyase as well as its reactivating factor, but they differ in presence and type of core metabolosome components, regulatory proteins, and genes that encode ancillary functions. Three features distinguish nearly every EUT2 locus from the EUT1 loci. First, while EUT2 loci usually encode a PduL-like PTAC, EUT1 loci uniquely among BMC loci encode the *pta*-like PTAC EutD (Fig. 4, Fig. S2). Additionally, nearly half of the EUT2 loci lack a complete metabolosome core; only EUT2A encodes the EutG AlcDH (Fig. 4). Moreover, in place of the EutR regulatory protein [82] found in EUT1 loci, a two-component regulatory system [83] is encoded in 87% of the EUT2 loci (Fig. S2). By contrast, EUT3 loci lack all of the metabolosome core enzymes but encode the signature enzyme subunits. *D. hafniense* also encodes two additional BMC loci which could contribute the AldDH and PTAC enzymes for the EUT BMC, but none of these loci encode an obvious AlcDH, indicating that EUT3 may not be a functional BMC or that the requisite AlcDH function is one encoded elsewhere in the genome.

Notably, LoClass detected several genes that have not previously been linked to ethanolamine utilization in 90% of EUT1 loci (Fig. 4, Dataset S1). For example, LoClass underscored a connection between the *eut* operon in EUT1 and a gene in the locus encoding malic enzyme MaeB, but its significance to the function of the EUT1 BMC is unknown, although there are hints of a connection. The C-terminus of MaeB contains a non-functional EutD-like PTAC domain [84]. MaeB is inhibited by acetyl-CoA [84], the substrate of the PTAC reaction. Also encoded in the EUT1 locus are the accessory proteins HemF coproporphyrinogen III oxidase and YfeX porphyrinogen oxidase [85], which may be involved in cobalamin metabolism. Only EUT2C loci lack the accessory protein EutT (cobalamin adenosyltransferase) and EutQ (a member of the cupin family of unknown function [86]), otherwise found in EUT1 and all other EUT2 locus sub-types (Dataset S1). Both EUT2B and 2D loci encode the PduS oxidoreductase, a flavoprotein, and an acetate kinase, which could convert acetyl-phosphate to acetate, generating ATP (Fig. 1B). Instead of the two-component regulatory system found in other EUT2 loci, EUT2D encodes a PocR domain-containing regulatory protein. Additional observations and comparisons of the various EUT locus (sub)types are included in Text S1.

### The Propanediol and Ethanolamine Utilizing (PDU/EUT) Fusion Loci (Cluster 2.1.1.2)

LoClass identified the first examples of fusions of BMC loci. With one exception (a PDU/GRM fusion, Dataset S1), these are combinations of various PDU and EUT loci. They are found primarily in the genus *Listeria*, as well as in the genomes of the actinobacterium *Propionibacterium* sp. oral taxon 192 str. F0372, the firmicute *Streptococcus sanguinis* SK36, and the fusobacterium *Sebaldella termitidis* ATCC 33386 (Table S2). This locus has been previously described in *Listeria* species [87]–[89]. Although the EUT portion of the fusion was assumed to be comparable to the canonical *eut* operon (EUT1 in the LoClass taxonomy), it is instead more closely related to EUT2A loci (Fig. 4). This is fused to a rearranged PDU1A locus that contains additional AlcDH genes at its terminus (Fig. 4). Despite the merging of the PDU1A and EUT2A loci in *Listeria*, there is no evidence to suggest that they are co-transcribed. Indeed, the regulatory protein PocR is present in the PDU region of the fusion locus, and the two-component regulatory proteins common to EUT2A loci are encoded in the EUT2 region (Fig. 4), suggesting that these are independently regulated. Both regions include ancillary genes related to cobalamin synthesis (Dataset S1).

The non-*Listeria* PDU/EUT loci are quite different from those of *Listeria* and from each other. The most significant examples are in *S. sanguinis* and *Propionibacterium*, where the order in which the PDU and EUT loci appear in the genome is inverted compared to the order in *Listeria* (Dataset S1), suggesting that they may have originated from fusion events distinct from that which generated the *Listeria* fusion locus.

Several selective pressures may potentially have driven the repeated fusion events of these two particular classes of loci, such as the benefit of coregulation with the cobalamin biosynthesis genes found in the merged locus, since both PDU and EUT require vitamin B_12_ as a cofactor. A PDU/EUT merger would be significantly more effective if the host organisms are commonly exposed to environments that contain both propanediol and ethanolamine. For example, the PDU and EUT loci have been implicated in improving the intracellular growth (and, as a result, virulence) of *S. enterica* [90] and *L. monocytogenes* [88], implying that propanediol and ethanolamine are relevant to pathogenesis. However, the PDU and EUT loci are separate in *S. enterica*, suggesting that other factors may have driven a fusion event in *L. monocytogenes*, such as evolutionary pressures for a reduced genome; *L. monocytogenes* has a genome of 3 Mbp, while *S. enterica* has a genome of almost 5 Mbp. Similar evolutionary forces may have been at play to fuse the PDU and EUT loci in the non-pathogenic strains.

### The Glycyl Radical Enzyme-Containing Microcompartment (GRM) Loci (Clusters 2.2–2.5, 4.2)

LoClass identified five distinct types of loci that contain the metabolosome core enzymes and a glycyl radical enzyme (Fig. S1), which, with its activating enzyme, we predict to be the signature enzymes of this class of metabolosome (Fig. 4). GRM loci are widespread, found in members of the phyla Actinobacteria, Firmicutes, Proteobacteria (Table S1), and are differentiated by their complement of shell proteins and accessory genes, such as regulators, transporters, and other genes that could encode ancillary functions.

The GRM1 locus (Cluster 2.2) is mainly found in the Firmicutes but also in some species of the Deltaproteobacteria and *Olsenella uli*, a member of the Actinobacteria (Table S2). This locus encodes one additional AldDH (Fig. 4, Dataset S1), suggesting that this metabolosome may degrade several different aldehydes. Accordingly, we aligned all AldDH sequences found in metabolosome loci to identify differences in the active site (Dataset S6). Surprisingly, we observed that in all GRM1 loci with two AldDHs, one of the genes contains a mutation in the catalytic cysteine to either a serine or proline, indicating that the enzyme cannot efficiently catalyze a dehydrogenation reaction [91], [92]. Catalytically defunct enzyme domains are likewise found in the carboxysome [37], [68], [93]. Frequently, nonfunctional enzyme domains act as scaffolds or regulators in various systems (reviewed in [94]); this second AldDH could have similar functions in the GRM1 loci.

Apart from the signature and core enzymes, other notable ancillary proteins in the GRM1 loci include homologs to PduS, PduV/EutP, EutQ, EutJ, a multidrug resistance (MDR) efflux transporter, and a predicted transcription factor (Dataset S1). In the PDU metabolosome, PduS is involved in the biosynthesis of vitamin B_12_, an essential cofactor for propionaldehyde dehydrogenase [79], [95], [96]. Its conservation in GRM1 loci is unexpected, as there are no B_12_-dependent enzymes present in the locus. PduS has an iron sulfur cluster-binding domain, as does PduT (a BMC-T shell protein) and the pair of proteins has been proposed to be involved in electron transport across the shell [35], [97]. The cysteine coordinating the 4Fe-4S cluster in PduT is conserved in the BMC-T protein in the GRM1 loci. Therefore, it is plausible that the PduS homolog could accept electrons from the PduT homolog, either for catalytic purposes, and/or for shuttling electrons out of the shell. PduV, EutP, EutQ, and EutJ are all proteins of unknown function but are conserved members of their respective Confirmed (PDU1 or EUT1) Loci. The glycyl radical enzyme of the GRM1 locus has been experimentally characterized; it is a choline lyase, producing trimethylamine and acetaldehyde [98], similar to the EUT BMC in which the signature enzyme produces ammonia and acetaldehyde [19], [99].

The GRM2 locus (Cluster 2.3) is only found in the Gammaproteobacteria and mainly in pathogens. The only ancillary genes encoded in this locus are three distinct transcription factors (with only one found in all GRM2 loci) and two multi-drug resistance proteins (Fig. 4). Due to their conservation within the GRM2 locus, the transporters are likely related to the function of the metabolosome, perhaps as transporters for substrate. Interestingly, this locus does not contain any genes for BMC-T shell proteins, while the other GRM loci do (Fig. 4), suggesting GRM2 metabolosomes differ from the other GRM BMCs in some aspect of metabolite flux across the shell. Although functional metabolosomes lacking BMC-T proteins are known [25], the majority of BMC loci (27 of 30 Candidate/Confirmed BMC Locus (sub)types; Fig. 4, Dataset S1) encode one or more BMC-T proteins.

The GRM3 locus (Cluster 2.4) is found in both innocuous and pathogenic species of the *Clostridium*, *Desulfosporosinus*, and *Oscillibacter* genera of the Firmicutes, as well as in various alpha- and gammaproteobacteria (Table S1, Table S2). Ancillary proteins encoded in the loci include acetate kinase, a peptidase, a flavoprotein, a EutJ homolog, S-adenosylmethionine synthetase, a pair of two-component signaling proteins, and a protein with a domain of unknown function (DUF336; Dataset S1). The potential role of a peptidase in the context of a BMC is unclear; perhaps it plays a regulatory role. The flavoprotein could be involved in a peripheral enzymatic step (including a possible role as an electron shuttle). S-adenosylmethionine (SAM) is a required cofactor for glycyl radical enzyme chemistry (reviewed in [100]), and the presence of SAM synthetase indicates that the locus encodes the accessory function of synthesizing SAM from ATP and methionine. Along with an adenosyltransferase domain, DUF336 is one of two domains found in PduO, an enzyme involved in the synthesis of vitamin B_12_ in the PDU BMC [101].

The GRM4 locus (Cluster 2.5) is restricted to two *Shewanella* species (Table S2). Without additional examples for comparison, we confined predictions of ancillary genes to those found within the region bounded by the core AlcDH and shell proteins (Fig. 4). In this set of genes, there is a major intrinsic protein (65.59% identity to the PduF transporter in *S. enterica*), as well as a protein containing the PduO component domain DUF336 (see above; Dataset S1).

The GRM5 locus (Cluster 4.2) is only found in firmicutes of the *Ruminococcus* genus, *Roseburia inulinivorans*, and *Clostridium phytofermentans* (Table S2). Ancillary genes within this locus consist of a transcriptional regulator in the DeoR family, a PduS homolog, a class II aldolase, a hydrolase, and a protein with DUF336 (Dataset S1). Transcriptional profiling of this locus suggests that its function is the anaerobic degradation of L-fucose and L-rhamnose, similar deoxy sugars [24], [102]. The aldolase is expected to cleave the hexose, one product being lactaldehyde that is further converted to propanediol, which the glycyl radical enzyme is expected to dehydrate to propionaldehyde [24]. The predicted AlcDH [24] in this locus contains the zinc-binding dehydrogenase pfams, which are different than the typical AlcDH pfam found in BMC loci (iron-binding), indicating that the specific type of a core component can be plastic as long as the function is maintained.

### Planctomycetes and Verrucomicrobia-Type (PVM) and PVM-Like Loci (Clusters 4.1 and 4.3)

One BMC locus (Cluster 4.1) is almost exclusively restricted to species in the phyla Planctomycetes and Verrucomicrobia (Table S1). The metabolosome encoded by this locus has recently been experimentally characterized; it is involved in the aerobic degradation of L-fucose and L-rhamnose [25]. Its signature enzyme is a class II aldolase (homologous to that in GRM5), and ancillary genes include a DeoR-family transcriptional regulator and an acetate kinase (Dataset S1). Again, the hypothesis that some plasticity is tolerated in the core enzyme composition is supported by the PVM BMC; a gene with the lactate/malate dehydrogenase pfams was predicted to provide core AlcDH function for the PVM BMC, as it is the only gene (other than AldDH) present in the locus that could regenerate NAD^+^ [25].

PVM-like loci (Cluster 4.2) are compositionally distinct yet cluster with the PVM loci (Fig. 3). The PVM-like loci are found in diverse members of the Firmicutes, the actinobacterium *Candidatus Solibacter usitatus*, and the ignavibacterium *Melioribacter roseus* (Table S2). A class II aldolase is found in each these loci, and a DeoR family transcriptional regulator is found in most, but only one encodes the core AlcDH (Dataset S1). The complement of shell protein genes is also peculiar; most of the PVM-like loci encode more BMC-H than BMC-P proteins; only *M. roseus* and one locus in *S. usitatus* have ratios characteristic of the PVM loci, which generally have more BMC-P than BMC-H proteins (Table S2). One locus completely lacks BMC-P genes, but this is one of two PVM-like loci in the *S. usitatus* genome; the other locus does encode BMC-P genes, and the gene products of the two loci could constitute a functional organelle (Dataset S1). Given that the GRM5 locus also contains an aldolase, these similarities in shell protein complement may indicate that most of the PVM-like loci are more closely related to the GRM5 loci, while the loci in *M. roseus* and *S. usitatus* could be more closely related to the PVM loci. Indeed, by comparing the genetic organization between the GRM5, PVM-like, and PVM representative loci in Fig. 4, the PVM-like representative locus is more syntenic to the GRM5 locus than to the PVM locus, where the gene order is completely different. Considering these observations, the PVM-like loci may be fragments of a related locus, perhaps GRM5.

### The Ethanol-Utilizing (ETU) Metabolosome Loci in *Clostridium kluyveri* (Cluster 2.7)

One BMC locus, present in two *Clostridium kluyveri* species, forms a distinct subgroup of Cluster 2 (Fig. S1). Although this locus fits our definition of a satellite locus, this set of genes has been implicated in ethanol degradation, where ethanol is oxidized to acetyl-CoA in two enzymatic steps [47], [103], [104]. The locus contains an incomplete metabolosome core, only encoding two AldDHs with identical sequences and three AlcDHs (Fig. 4), which can assemble into an aldehyde-alcohol dehydrogenase complex [103], [104]. The proposed biochemical pathway for ethanol utilization, ethanol to acetaldehyde to acetyl-CoA, consumes two NAD^+^ and one CoA. However, it is not obvious how these cofactors are recycled; the locus lacks a PTAC, and using any of the AlcDHs to recycle NAD^+^ would result in a futile cycle. It is appealing to solve this conundrum by positing that a BMC shell is not formed, but polyhedral structures have been observed in this species when fermenting ethanol and acetate [105]. Interestingly, the locus also lacks genes for BMC-P proteins, which are required to complete the diffusional barrier formed by the shell; shells deficient in BMC-P proteins are more “leaky” [18], [28]. It may be that the ETU BMC shell is open enough that cofactor recycling within the organelle is not required. Although a leaky shell may not effectively sequester acetaldehyde, there may be another benefit to spatially clustering the enzymes, such as substrate channeling. Alternatively, genes from the PDU-like locus present in this species (Fig. 4; see above) could be co-opted, providing the BMC-T, BMC-P, and PTAC genes to form a complete metabolosome.

### Metabolosomes of Unknown Function (RMM, MUF, MIC; Clusters 2.6, 5, 2.1.2, and 7)

In addition to Candidate/Confirmed Loci that we could classify and for which we could infer putative BMC-related reactions, we also identified five locus types for which we could not easily predict a function. Four of these are distinctly different from metabolosomes in that they do not encode a complete biochemical core. Two of these locus types are found exclusively in members of the phylum Actinobacteria – specifically in several species of the genus *Mycobacterium* and in *Rhodococcus jostii* (Table S2); accordingly, we refer to these as the *Rhodococcus* and *Mycobacterium* Microcompartment (RMM) loci. Two more locus types were observed in phylogenetically distinct organisms; these are designated Metabolosomes of Unknown Function (MUF) or Metabolosomes with an Incomplete Core (MIC).

The major difference between the two types of RMM loci is the presence (RMM2; Cluster 2.1.2) or absence (RMM1; Cluster 5) of diol dehydratase genes (Fig. 4). Putative ancillary proteins encoded in the RMM1 locus include multiple hydrolases, a phosphotransferase, a short chain dehydrogenase, an amino acid permease, and a regulatory protein of the GntR family (Dataset S1). Amino-2-propanol has been shown to be an inducer of the short chain dehydrogenase via GntR [106], and the short chain dehydrogenase has been shown to convert amino-2-propanol to aminoacetone [107]. If the aminotransferase were to act on aminoacetone, methylgyloxal would be produced, which is extremely toxic (reviewed in [108]). This toxicity could be alleviated by conversion of methylglyoxal to pyruvyl-CoA by the core AldDH. However, as mentioned above, both the NAD^+^ and CoA used in this step must be regenerated somehow within the compartment, and the core metabolosome genes to do so are not obviously present. Perhaps the aforementioned ancillary enzymes form a circuit that recycles the required cofactors. For example, a hydrolase could regenerate CoA to form lactate. Alternatively, it may be that some BMC shells allow cofactors across, or that the AldDH may not be acylating, at least not requiring CoA to be recycled.

The RMM2 locus contains the same set of genes as RMM1 but has several additional genes and diol dehydratase homologs, while the hydrolases present in RMM1 are absent (Dataset S1). Based on their annotations, none of the constituent genes appear to catalyze the regeneration of NAD^+^ or CoA. The subset of genes in the locus spanning from the permease to the transcription factor are highly syntenic between RMM1 and RMM2 (Fig. 4), suggesting that RMM1 and RMM2 metabolosomes function similarly but have different peripheral reactions. Furthermore, the diol dehydratase genes are on the opposite strand from these ancillary genes and would not be on the same polycistronic message as the rest of the genes in the locus. We interpret these observations to indicate that the RMM2 BMC does not utilize the diol dehydratase genes and is instead involved in amino alcohol degradation. Alternatively, the BMC may be bifunctional, capable of using both the diol dehydratase and aminotransferase as signature enzymes.

The MUF locus (Cluster 2.6) is present in *Clostridium botulinum* B str. Eklund 17B and *Clostridium botulinum* E3 str. Alaska E43 (Table S2) and includes all metabolosome core enzymes but lacks a prospective signature enzyme (Fig. 4). This locus also encodes BMC-H, BMC-T, and BMC-P as well as PduS- and EutJ-like proteins (Dataset S1). These observations indicate that MUF is likely a functional metabolosome, possibly involved in degradation of an unspecified aldehyde, or that its signature enzyme is recruited from elsewhere in the genome.

The MIC1 (Cluster 7) metabolosome locus contains a dehydrogenase in addition to AldDH and two putative AlcDHs, as well as a hydrolase, a phosphatase, and an MreB-like protein (Dataset S1), but lacks a PTAC and a signature enzyme, which may be encoded elsewhere in the genome. One dehydrogenase contains the lactate/malate dehydrogenase pfams, similar to the predicted core AlcDH in the PVM locus, while one contains the zinc-binding dehydrogenase pfams found in the GRE5 locus, so we could not resolve which provides the core recycling function. Due to the low number of conserved genes in the locus, it is not possible to predict how CoA is recycled. However, as for the RMM loci, there are possible alternatives for circumventing this necessity (discussed above).

Four additional MIC loci (Dataset S1), each with incomplete metabolosome cores, were identified, and there is only one representative of each. These include loci from the actinobacterium *Verrucosispora maris* AB-18-032 and the firmicute *Mahella australiensis* 50-1 BON, each of which encodes a class II aldolase, which could serve as the signature enzyme (Dataset S1). Other MIC loci are found in the deltaproteobacterium *Haliangium ochraceum* and in *Synergistetes bacterium* SGP1 (Dataset S1). Based on the presence of an AldDH, the RMM and MIC BMCs likely encapsulate an aldehyde, but the absence of AlcDH and/or PTAC to recycle CoA and NAD^+^ indicates cofactor recycling is not absolutely required. If this hypothesis is correct, these BMCs would be inaugural members of a new class of aldehyde-processing metabolosomes.

### BMC Loci from Candidate Phyla Including Single-Cell Genomes

We also identified loci found in candidate phyla mostly comprised of incomplete single-cell genomes and compared them to the BMC taxonomy (Fig. 4). Many of these loci are incomplete, occurring at the end of a scaffold. However many fragments are long enough to show synteny to other loci of the same type and to differentiate them from Candidate/Confirmed BMC Loci identified in NR (Dataset S2). The majority of these divide into two main types, discussed below.

One Candidate BMC Locus type found in single-cell genomes belonging to the *Atribacteria*, *Gemmatimonadetes*, and *Marinimicrobia* genera (Fig. 6, Dataset S2) contains an aldolase 52–57% identical to DeoC deoxyribose-phosphate aldolase (EC: 4.1.2.4; UniProtKB: Q9X1P5), a class I aldolase that cleaves 2-deoxyribose 5-phosphate into glyceraldehyde-3-phosphate and acetaldehyde [109]. These loci also contain an AldDH, possibly forming acetyl-CoA from acetaldehyde. Moreover, all but *Marinimicrobia* contain a PduL PTAC (Dataset S2). All of these loci contain a sugar isomerase 48–53% identical to RpiB ribose-5-phosphate isomerase (EC: 5.3.1.6; UniProtKB: A3DIL8), which isomerizes ribose-5-phosphate to ribulose-5-phosphate [110], [111]. A number of RpiB proteins exhibit low substrate specificity, isomerizing a wide variety of sugars [112]. In *Marinimicrobia*, the aldolase and isomerase occur as a fusion protein.

Of these loci, only those in *Atribacteria* contain a PduS homolog, the oxidoreductase associated with cobalamin biosynthesis. However, cobalamin is not a required cofactor for DeoC type aldolases. As AlcDH is the only core enzyme absent from these loci, it is possible that PduS may generate an alcohol from an aldehyde, as predicted in one GRM5 locus [24]. PduS also may be present in these loci as a remnant of common ancestry with the *pdu* operon.

The *Atribacteria bacterium* JGI 0000079-F20 locus additionally encodes a protein 44% identical to triosephosphate isomerase (TIM) (EC: 5.3.1.1; UniProtKB: P00943), which catalyzes the reversible conversion of glyceraldehyde-3-phosphate to dihydroxyacetone phosphate. When acted on by an aldolase, sugar phosphates can be metabolized to either or both of these products, in some cases resulting in another product such as an aldehyde, which could be metabolized according to the canonical metabolosome model. Thus, based on the presence of TIM and a DeoC-like aldolase, we name these BMCs putative sugar phosphate utilizing (SPU) metabolosomes with an incomplete core; most of the enzymes encoded in the SPU loci fit into the deoxyribulose/deoxyribose 5-phosphate degradation pathway for DNA catabolism.

The second Candidate BMC Locus type that appears in single-cell genomes is found in two representatives from the candidate phylum Latescibacteria. This locus is similar to the SPU type, containing genes encoding an AldDH, PduL, PduS, an RpiB-like protein, and an aldolase (Dataset S2), so we refer to it as SPU-like. Interestingly, the aldolase is different than in SPU; instead, a class II aldolase like those found in several other BMC loci is present. Despite this difference, the enzyme could still generate an aldehyde from a sugar phosphate as in the GRM5 and SPU locus types. In addition, this locus contains a protein 40–44% identical to acetate kinase (EC: 2.7.2.1; UniProtKB: Q9WYB1). Neither the SPU nor SPU-like loci had been observed prior to the analysis of these single-cell genomes from candidate phyla, indicating that with continued sequencing of microbial “dark matter” [52] many more BMC loci types may be discovered.

Several additional Candidate and satellite-like loci found in single-cell genomes could only be classified as metabolosome loci with incomplete cores. These were identified in members of the candidate phyla Caldithrix, Poribacteria [113] and BRC1 (Dataset S2). *Caldithrix abyssi* contains a locus which encodes AldDH, a *pta* type PTAC, and PduS. However, the absence of an apparent signature enzyme necessitates the designation of this locus as a MIC. Each sequenced locus from Poribacteria and BRC1 meets all of the criteria of a satellite-like locus (Dataset S2) which may indicate that the region of the genome encoding the core locus has not been sequenced, or that BMC-related genes are constitutively expressed and so are not required to be proximal to other BMC-related genes; several of these satellite loci encode a PduL PTAC or an AldDH, indicating that these BMCs may function as metabolosomes.

### Validation of the LoClass Taxonomy: The Molecular Phylogeny of BMC-Associated AldDH

Based on our observation that the majority of heterotrophic BMC loci encode an AldDH (Fig. 4, Fig. S2), we hypothesized that these protein sequences may be used to construct a phylogeny of this large subset of BMCs, which could be used to validate the LoClass method. We excluded the presumably catalytically defunct AldDHs found in the GRM1 loci (discussed above), but we did include other duplicate AldDHs that were not obviously altered in any catalytic residues and could remain functional (i.e. we could not resolve which one was the core AldDH).

In the resulting phylogenetic tree, we observed two major “trunks” that were separated by a relatively long branch (Fig. 5A) and rooted the tree at this branch (Fig. 5B). All PDU, RMM, PVM/PVM-like, and SPU/SPU-like associated AldDHs cohere with their respective trunks, while the other BMC (sub)types do not initially seem as polarized; sequences from EUT and GRM loci were found on both trunks (Fig. 5B). However, when mapping LoClass's sub-clustering results onto the phylogeny, we observed EUT and GRM subgroups to also display bifurcated localization: EUT1, GRM3, GRM4, and GRM5 sequences predominantly localized to one trunk, while EUT2, GRM1, and GRM2 sequences clustered on the other trunk. Furthermore, sequences from most metabolosome loci formed clades corresponding to the locus (sub)types shown in Figures 3 and 4 (Fig. 5B). The only major exceptions to this are two GRM1 taxa that branch close to EUT1, two GRM3 taxa that branch close to the AldDH from the PDU/EUT locus in *Listeria*, and one GRM3 sequence adjacent to ETU taxa (asterisks in Fig. 5). The outlier GRM1 sequences belong to a second GRM1 locus in their respective organisms (*Desulfotalea psychrophila* LSv54, *Desulfitobacterium hafniense* DCB-2); this may indicate that this additional locus has a distinct function. The GRM3 outliers near the PDU/EUT sequences are secondary AldDH genes in their respective loci (*Clostridium* cf. *saccharolyticum*, *Clostridium beijerinckii*). The GRM3 AldDH near the ETU branch belongs to *Clostridium novyi*; its proximity to ETU may indicate an evolutionary link between GRM3 and ETU. In addition, some GRM3 taxa were found nested within the large GRM1 clade, which may indicate that they share common origin with GRM1 loci. Thus, apart from these few outlier taxa, we observed congruence between the molecular phylogeny of one gene (AldDH) and our clustering results based on total pfam complement of a locus (Fig. 3, Fig. S1), validating our LoClass-based metabolosome taxonomy.

### BMCs of Unknown Function (BUF; Cluster 6): A Third BMC Paradigm?

In contrast to all other metabolosome loci, including MIC loci, Cluster 6, found only in four different species of Firmicutes, lacks all metabolosome core enzymes but does encode BMC-H, BMC-T, and BMC-P genes (Fig. 4), suggesting that a complete shell is formed. Accordingly, we refer to this locus type as BMC of Unknown Function (BUF). Many of the genes encoded within the BUF locus are putative enzymes, including amidohydrolases, deaminase, dehydrogenase, carboxy-lyase, carbon monoxide hydrogenase, isochorismatase, and formiminotransferases (Dataset S1), making it difficult to predict a function. However, the presence of amidohydrolases and deaminases suggests that nitrogen-containing compounds are processed via this Candidate BMC Locus. Alternatively, it is possible that this BMC encapsulates enzymes from the core metabolic model that are scattered throughout the genome. However, since the enzymes in the locus are conserved across these loci, and no other loci besides the carboxysome maintain a conserved locus while lacking all core metabolosome enzymes, these observations, and notably the absence of the core AldDH pfam, suggest that this BMC encapsulates a distinctly different metabolite. This locus appears to encode a new type of BMC that does not conform to the metabolosome or carboxysome functional paradigms.

### Summary and Prospects

Methods for detection and classification of conserved loci typically approach the problem with the goal of operon detection [114], [115], require analysis of multiple whole genomes [115]–[117], are sensitive to genomic rearrangements and gene order [114], [116], or through computationally-driven mergers produce extended gene clusters that may not exist in any one genome [116]. Even after a conserved gene cluster is identified, none of these methods enable the direct comparison and classification of these loci by an automated scoring method. LoClass circumvents all of these issues. By “seeding” with BMC shell protein genes, we confined the scope of our analysis to genomic regions most likely to encode BMC-related functions. Then, based on the assumption that genes encoding other BMC structural components and supporting functions would be proximal to the shell protein genes, as in experimentally characterized BMCs, we were able to compare loci based entirely upon the subset of constituent genes that did not encode BMC shell proteins. Because this assumption will likely be valid for other gene clusters encoding macromolecular complexes or metabolic pathways, the LoClass strategy may be useful for detecting contiguous groups of genes that functionally cohere.

LoClass enabled us to compare and classify hundreds of BMC loci. Moreover, it allowed detection of functional sub-types, prediction of novel BMC types, and identification of genes that support the function of the BMC that are not structurally part of the organelle. Among these ancillary pfams are, for example, actin-like proteins (PF06723 and PF11104) and ParA (PF01656) (Fig. 4, Dataset S1). MreB, a bacterial actin, and ParA have been implicated in spatial localization of carboxysomes [73]. PduV has also been suggested to play a role in the spatial positioning of the PDU BMC [118], and we found homologs (containing PF10662) to be encoded in most PDU, EUT, and GRM1 loci, as well as in a quarter of all satellite-like loci. The frequent observation of these pfams in BMC loci suggests that spatial positioning within the cell is important to the formation and function of BMCs. Likewise, we found that many Candidate/Confirmed BMC Loci contain genes for transporters; their distinctive types suggest that they may be specific to the substrate(s) processed by the BMC. Regulatory genes were also frequently observed; for example, EUT2D, some GRM1, GRM3, GRM4, and ETU loci, in addition to the PDU loci, encode regulatory proteins that contain the PocR domain. Considering that all experimentally characterized metabolosomes are only induced in the presence of their substrate, these putatively BMC-associated genes are not likely to be “genomic hitchhikers,” genes unrelated to the function encoded by the locus but merely similarly transcriptionally regulated [116]; rather, they are likely genes that provide important supporting functions for the BMC. Recognition of these cryptically associated accessory genes should prove useful in efforts to functionally characterize diverse BMCs.

In addition to expanding the vocabulary of pfams associated with bacterial organelle function, LoClass unveiled BMC-associated puzzles, pfams for which we could not readily infer a reason for their conservation in various locus types. For example, GRM1, GRM3, GRM4, GRM5, MUF, SPU, and SPU-like loci, which do not encode cobalamin-dependent enzymes, encode various genes that have been associated with cobalamin synthesis, such as PduS and PduO (both full length and a single DUF336 domain). These may be relics of the evolutionary history of these BMC loci or, more likely, these genes have been co-opted to perform different functions useful to the BMC metabolism, as has been predicted in the GRM5 locus in *C. phytofermentans* [24]. On the other hand, the conserved co-localization of the light-dependent and light-independent protochlorophyllide reductase genes with the cyanobacterial alpha-carboxysome loci and of the MaeB malic enzyme with EUT1 loci cannot yet be explained. One intriguing trend uncovered by LoClass is the frequent presence of domains associated with flavin binding or utilization (PF02441, PF01593, PF03358, PF00941, PF01494, and PF00258; Dataset S1). Furthermore, PduS has been shown to bind flavin mononucleotide [95], [96]. The frequent presence of flavin-binding pfams and proteins in BMC loci suggests that these cofactors could play a previously unrecognized and important role in BMC biochemistry and redox.

LoClass also highlighted the diversity within numerically abundant locus types (Fig. 4); this has only been attempted previously for EUT loci [119]. Additionally, capturing genes for functions ancillary to the BMC and their contribution to the clustering led to detection of unanticipated relationships. A striking example of this is seen when comparing the carboxysome loci. Due to the low similarity between the core structural components (i.e. between the encapsulated carbonic anhydrases/CcmN and CsoS2) of the alpha- and beta-carboxysome, it was unexpected that they would cluster together. Investigation revealed that the principle cause of this clustering is the NDH-1_3_/NDH-1_4_ CO_2_ transport gene cluster, which is conserved across cyanobacterial carboxysome loci of both types.

Several striking observations from our classification enlarge our view of the diversity of BMCs. New locus types identified in this study are the PDU/EUT and PDU/GRM fusions, GRM2, GRM3, GRM4, PVM-like, RMM2, MUF, MIC1, SPU, SPU-like, and BUF locus types. While many of these BMC loci adhere to the complete biochemical model of the metabolosome paradigm (aldehyde utilization and the regeneration of cofactors, catalyzed by a PTAC [32] and an AlcDH [31]), other metabolosome loci (those containing AldDH but missing PTAC and/or AlcDH) apparently regenerate or acquire cofactors in as yet unknown ways. LoClass also enabled us to identify new types of metabolosomes (e.g. multiple types of GRM loci). Furthermore, LoClass identified the BUF locus, a novel BMC type that does not conform to either the carboxysome or metabolosome paradigms, presenting a potentially new model of BMC metabolism. The novel locus types we identified are typically found in poorly characterized clades of the Bacterial domain, hinting that there are bacterial organelles of unknown function yet to be discovered.

Likewise, the observation of GRM, PVM, PVM-like, MIC, and SPU-like loci in diverse phyla that nevertheless contain homologous aldolases is interesting, given that class II aldolases are generally quite promiscuous [120]. Considering the presumed promiscuity of the core enzymes of metabolosomes, their colocalization with the class II aldolase suggests that these loci may be descendants of a common ancestor, one with a functionally malleable core due to substrate ambiguity of its component enzymes; this would render it readily able to confer new catabolic capabilities by horizontal gene transfer.

In addition to providing evolutionary insights, this descriptive, domain-based taxonomy of BMC loci is a guide to uncovering the functional diversity of BMCs and their roles as modules of metabolic specialization in bacteria; this parallels the historical discovery of eukaryotic organelles, in which observation and description laid the foundation for experimental elucidation of function. Domains are the structural, functional, and evolutionary units of proteins; analogously, LoClass captures (re)combinations of groups of domains that constitute loci and contribute to BMC function and evolution. Such a comprehensive view of the requisite building blocks for diverse BMC functions can likewise inform the design of BMC loci for the introduction of genetic and metabolic modules for applications in synthetic biology.

## Supporting Information

Dataset S1 **Gene information for all BMC loci in NR analyzed with LoClass.**
(XLSX)

Dataset S2 **Gene information for all BMC loci from candidate phyla examined in IMG.**
(XLSX)

Dataset S3 **Seed alignment for modified PF00936 domain.**
(FASTA)

Dataset S4 **Modified PF00936 domain HMM.**
(HMM)

Dataset S5 **Cytoscape session file for BMC locus similarity network.**
(CYS)

Dataset S6 **Aldehyde dehydrogenase alignment.**
(FASTA)

Figure S1 **Similarity network of BMC loci at all clustering levels.** All stages of clustering beyond the first stage are represented in this figure, where above each cluster is written its number. Clustering was accomplished by setting score (S) cut-offs and modifying the inflation value (I), a property of MCL [49]. The first level of clustering (I:2; S:3) resulted in ten clusters (Fig. 3). Cluster 1 was then further sub-clustered (I:3; S:10) to form three clusters. Cluster 2 was sub-clustered (I:2.5; S:10), resulting in seven clusters. We sub-clustered (I:3; S:20) Cluster 2.1, resulting in three sub-clusters. Then, we sub-clustered (I:3; S:40) Cluster 2.1.1 to form five sub-clusters. Cluster 3 was also sub-clustered (I:2; S:15) to form three sub-clusters. Subsequently, we sub-clustered (I:3; S:25) Cluster 3.2 to form four sub-clusters. Finally, Cluster 4 was sub-clustered (I:3; S:10), resulting in three clusters. Nodes represent loci containing bacterial microcompartment genes which were not predicted to be satellite loci. Edge length is proportional to the pairwise locus similarity score generated by LoClass. Node sizes are proportional to the number of genes in the envelope, the maximal region in the locus bounded by BMC genes. Node colors and shapes correspond to the locus (sub)type as predicted by our analysis and correspond to the key. The white circle in Cluster 1.3 indicates a locus in a synthetic genome not included in our analysis [121].
(TIF)

Figure S2 **Heatmap of common pfam occurrence in BMC locus (sub)types.** The 75 pfams present in the highest number of Candidate/Confirmed BMC Loci from NR and IMG are sorted by percentage of these loci that contained them. For each locus (sub)type, the proportion of loci of that given (sub)type that contain a specific pfam is represented by the darkness of the corresponding square, where black indicates that all of the loci contain that pfam and white indicates that none of the loci contain the pfam. Only locus (sub)types with more than one locus are shown. * Many of these loci are incomplete, and percent abundances may not reflect the percent of loci in these organisms that contain the corresponding pfam.
(TIF)

Figure S3 **Similarity network of BMC loci colored by phylum.**
(TIF)

Table S1 **Phyla containing BMC loci analyzed with LoClass.**
(DOC)

Table S2 **Genomes containing BMC loci analyzed with LoClass.**
(DOC)

Text S1 **Genes and pfams associated with the PDU and EUT loci.**
(DOCX)
