# Supplementary figures and images for: A Taxonomy of Bacterial Microcompartment Loci Constructed by a Novel Scoring Method
Source: PLoS Comput Biol. 2014 Oct 23;10(10):e1003898. doi: 10.1371/journal.pcbi.1003898 (PMC4207490; doi:10.1371/journal.pcbi.1003898)

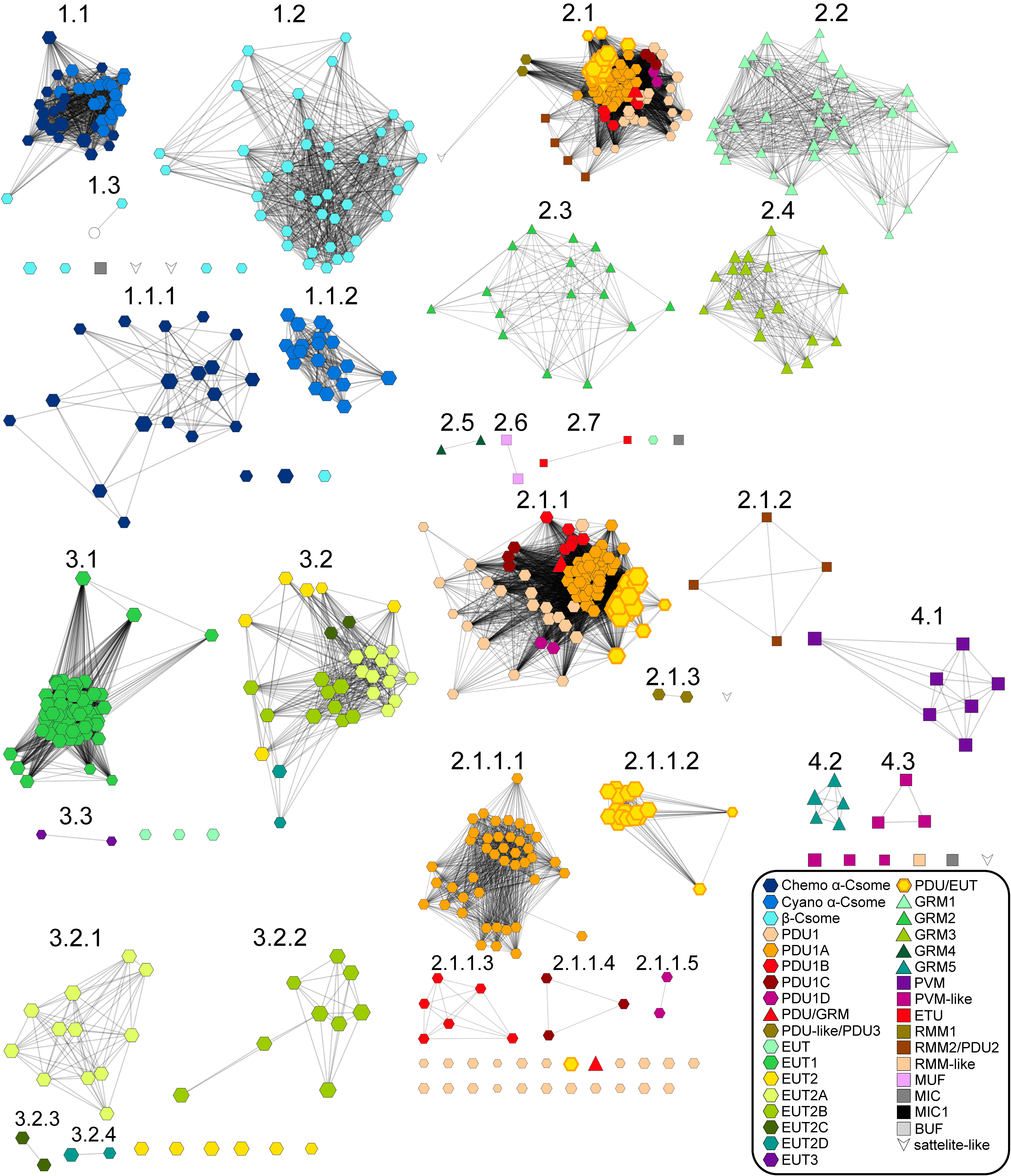

Supplement: Figure S1 — Similarity network of BMC loci at all clustering levels. All stages of clustering beyond the first stage are represented in this figure, where above each cluster is written its number. Clustering was accomplished by setting score (S) cut-offs and modifying the inflation value (I), a property of MCL [49]. The first level of clustering (I:2; S:3) resulted in ten clusters (Fig. 3). Cluster 1 was then further sub-clustered (I:3; S:10) to form three clusters. Cluster 2 was sub-clustered (I:2.5; S:10), resulting in seven clusters. We sub-clustered (I:3; S:20) Cluster 2.1, resulting in three sub-clusters. Then, we sub-clustered (I:3; S:40) Cluster 2.1.1 to form five sub-clusters. Cluster 3 was also sub-clustered (I:2; S:15) to form three sub-clusters. Subsequently, we sub-clustered (I:3; S:25) Cluster 3.2 to form four sub-clusters. Finally, Cluster 4 was sub-clustered (I:3; S:10), resulting in three clusters. Nodes represent loci containing bacterial microcompartment genes which were not predicted to be satellite loci. Edge length is proportional to the pairwise locus similarity score generated by LoClass. Node sizes are proportional to the number of genes in the envelope, the maximal region in the locus bounded by BMC genes. Node colors and shapes correspond to the locus (sub)type as predicted by our analysis and correspond to the key. The white circle in Cluster 1.3 indicates a locus in a synthetic genome not included in our analysis [121]. (TIF) [file pcbi.1003898.s007.tif]

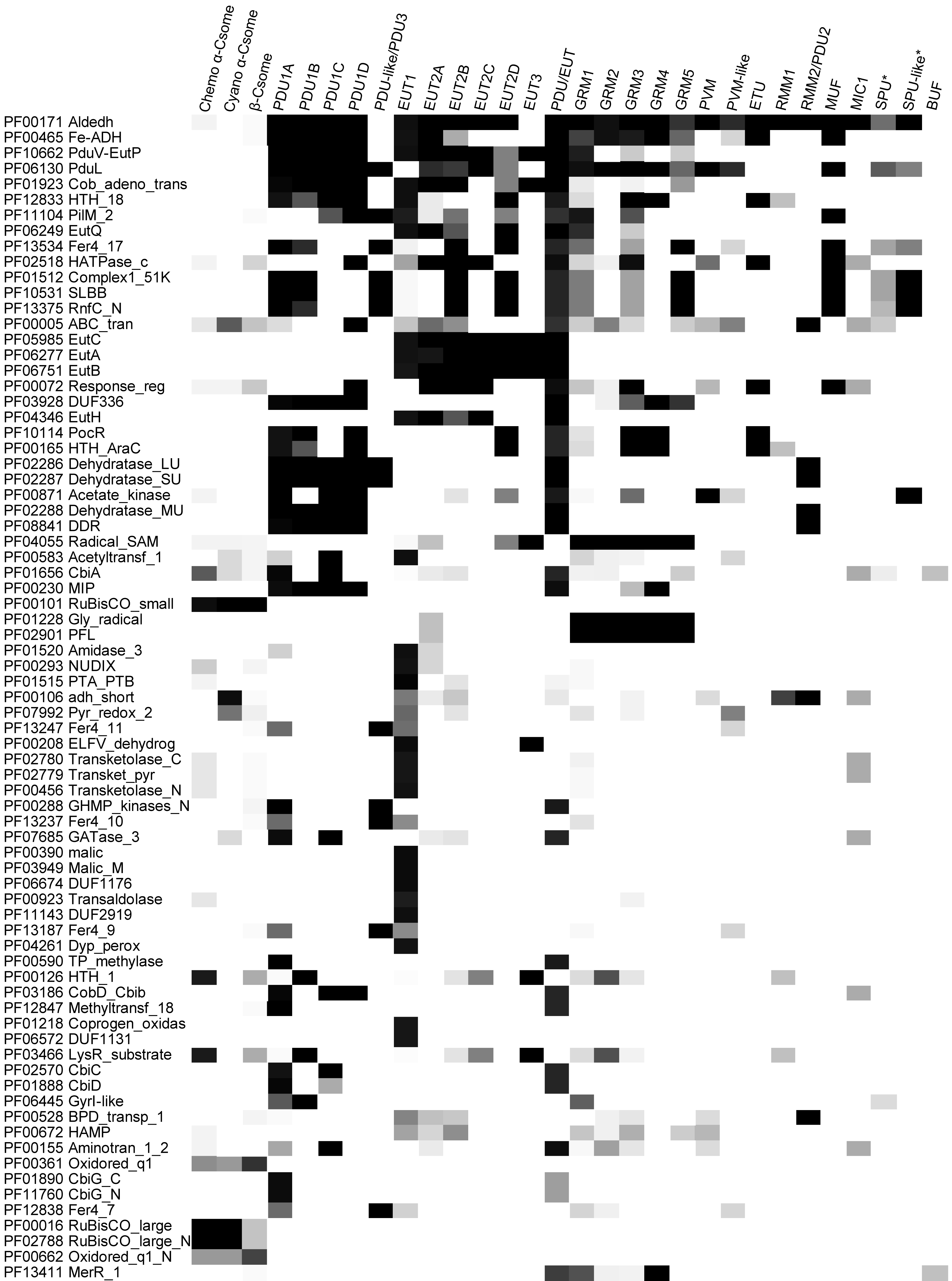

Supplement: Figure S2 — Heatmap of common pfam occurrence in BMC locus (sub)types. The 75 pfams present in the highest number of Candidate/Confirmed BMC Loci from NR and IMG are sorted by percentage of these loci that contained them. For each locus (sub)type, the proportion of loci of that given (sub)type that contain a specific pfam is represented by the darkness of the corresponding square, where black indicates that all of the loci contain that pfam and white indicates that none of the loci contain the pfam. Only locus (sub)types with more than one locus are shown. * Many of these loci are incomplete, and percent abundances may not reflect the percent of loci in these organisms that contain the corresponding pfam. (TIF) [file pcbi.1003898.s008.tif]

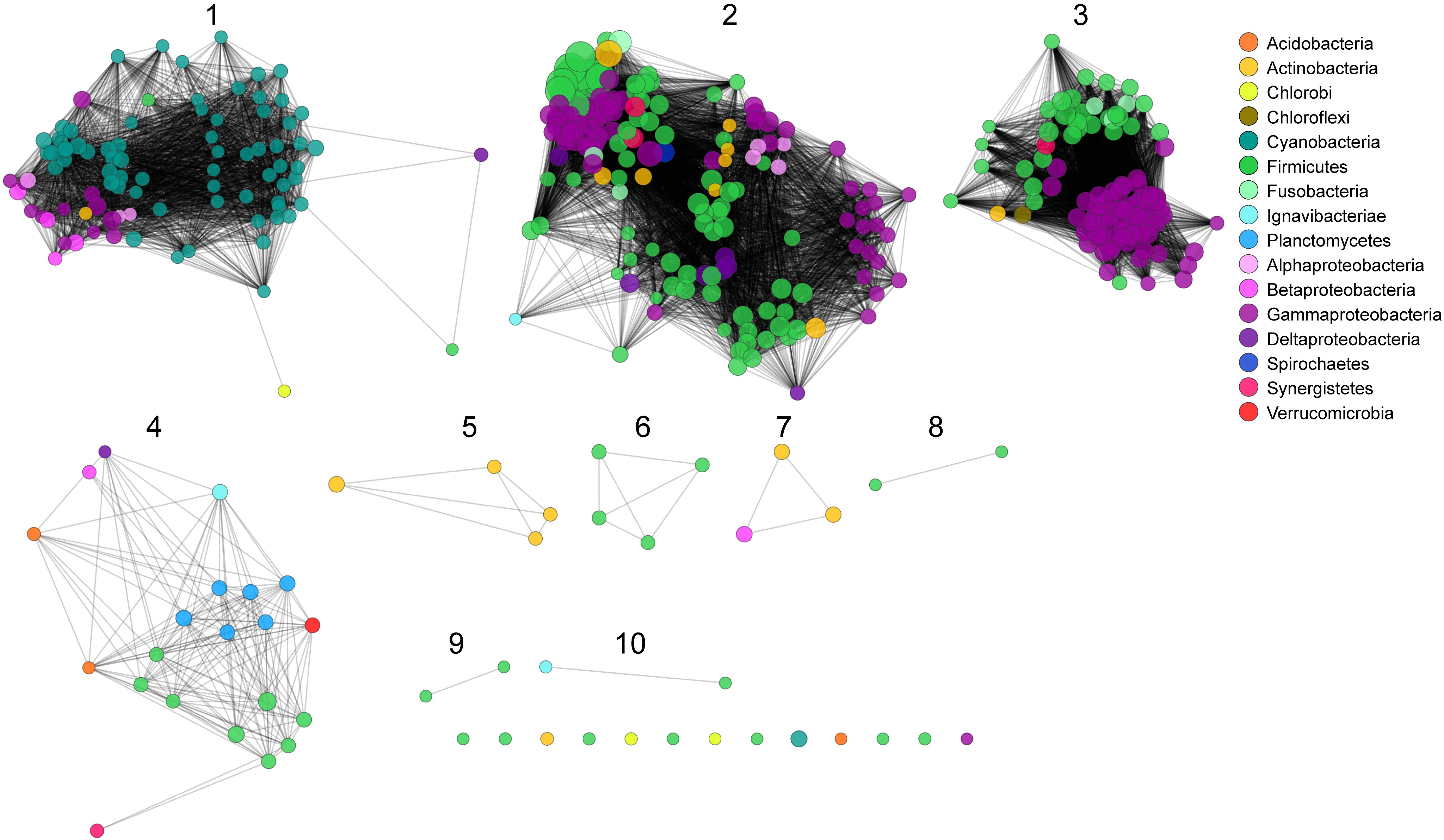

Supplement: Figure S3 — Similarity network of BMC loci colored by phylum. (TIF) [file pcbi.1003898.s009.tif]
