# Supplementary material for: A Taxonomy of Bacterial Microcompartment Loci Constructed by a Novel Scoring Method
Source: PLoS Comput Biol. 2014 Oct 23;10(10):e1003898. doi: 10.1371/journal.pcbi.1003898 (PMC4207490; doi:10.1371/journal.pcbi.1003898)
Supplement: Table S1 — Phyla containing BMC loci analyzed with LoClass. (DOC) [file pcbi.1003898.s010.doc]

**Table S1. Phyla containing BMC loci analyzed with LoClass.**

| **Phylum** | **Genomes Containing BMC Loci** | **All Predicted Locus (Sub)Types** |
| --- | --- | --- |
| Acidobacteria | 1 | PVM-like |
| Actinobacteria | 17 | Alpha-Carboxysome, EUT (unclustered), GRM1, MIC1, MIC (unclustered), PDU/EUT, PDU1 (unclustered), RMM1, RMM2/PDU2 |
| Chlorobi | 1 | all satellite-like |
| Chloroflexi | 1 | EUT1 |
| Cyanobacteria | 66 | Beta-Carboxysome, Alpha-Carboxysome |
| Firmicutes | 99 | BUF, ETU EUT2A, EUT2B, EUT2C, EUT2D, EUT2 (unclustered), EUT3, EUT (unclustered), GRM1, GRM3, GRM5, MIC (unclustered), MUF, PDU/EUT, PDU-like/PDU3, PDU1C, PDU1D, PDU1 (unclustered), PVM-like |
| Fusobacteria | 5 | EUT2A, PDU/EUT, PDU1 (unclustered) |
| Ignavibacteriae | 2 | all satellite-like, PVM-like |
| Planctomycetes | 6 | PVM |
| Alphaproteobacteria | 7 | Alpha-Carboxysome, GRM3 |
| Betaproteobacteria | 6 | Alpha-Carboxysome, MIC1, RMM-like |
| Gammaproteobacteria | 105 | all satellite-like, Alpha-Carboxysome, EUT1, EUT2B, GRM2, GRM3, GRM4, PDU/GRM, PDU1A, PDU1B, PDU1 (unclustered) |
| Deltaproteobacteria | 7 | GRM1, MIC (unclustered), PDU1 (unclustered) |
| Spirochaetes | 1 | PDU1 (unclustered) |
| Synergistetes | 4 | EUT2B, MIC (unclustered), PDU1 (unclustered) |
| Verrucomicrobia | 1 | PVM |
