# Supplementary material for: A Taxonomy of Bacterial Microcompartment Loci Constructed by a Novel Scoring Method
Source: PLoS Comput Biol. 2014 Oct 23;10(10):e1003898. doi: 10.1371/journal.pcbi.1003898 (PMC4207490; doi:10.1371/journal.pcbi.1003898)
Supplement: Table S2 — Genomes containing BMC loci analyzed with LoClass. (DOC) [file pcbi.1003898.s011.doc]

**Table S2. Genomes containing BMC loci analyzed with LoClass.**

| **NCBI Taxon ID** | **Phylum** | **Organism Name** | **Total Locus Count** | **Predicted Satellite Locus Count** | **Satellite-like Locus Count** | **BMC-H Count** | **BMC-T Count** | **BMC-P Count** | **All Predicted Locus (Sub)Types** |
| --- | --- | --- | --- | --- | --- | --- | --- | --- | --- |
| 234267 | Acidobacteria | *Candidatus Solibacter usitatus* Ellin6076 | 5 | 2 | 1 | 3 | 3 | 3 | PVM-like |
|  |  |  |  |  |  |  |  |  |  |
| 525909 | Actinobacteria | *Acidimicrobium ferrooxidans* DSM 10331 | 1 | 0 | 0 | 3 | 0 | 1 | Alpha-Carboxysome |
| 710421 | Actinobacteria | *Mycobacterium chubuense* NBB4 | 1 | 0 | 0 | 1 | 2 | 1 | RMM2/PDU2 |
| 350054 | Actinobacteria | *Mycobacterium gilvum* PYR-GCK | 1 | 0 | 0 | 1 | 2 | 1 | RMM2/PDU2 |
| 278137 | Actinobacteria | *Mycobacterium gilvum* Spyr1 | 1 | 0 | 0 | 1 | 2 | 1 | RMM2/PDU2 |
| 246196 | Actinobacteria | *Mycobacterium smegmatis* str. MC2 155 | 1 | 0 | 0 | 1 | 2 | 1 | RMM1 |
| 164757 | Actinobacteria | *Mycobacterium* sp. JLS | 1 | 0 | 0 | 1 | 2 | 1 | RMM1 |
| 164756 | Actinobacteria | *Mycobacterium* sp. MCS | 1 | 0 | 0 | 1 | 2 | 1 | RMM1 |
| 350058 | Actinobacteria | *Mycobacterium vanbaalenii* PYR-1 | 1 | 0 | 0 | 1 | 2 | 1 | RMM2/PDU2 |
| 479431 | Actinobacteria | *Nakamurella multipartita* DSM 44233 | 1 | 0 | 0 | 2 | 1 | 1 | MIC1 |
| 196162 | Actinobacteria | *Nocardioides* sp. JS614 | 1 | 0 | 0 | 1 | 2 | 1 | EUT (unclustered) |
| 633147 | Actinobacteria | *Olsenella uli* DSM 7084 | 1 | 0 | 0 | 8 | 0 | 1 | GRM1 |
| 754252 | Actinobacteria | *Propionibacterium freudenreichii* subsp. *shermanii* CIRM-BIA1 | 1 | 0 | 0 | 4 | 1 | 1 | PDU1 (unclustered) |
| 767029 | Actinobacteria | *Propionibacterium propionicum* F0230a | 1 | 0 | 0 | 4 | 1 | 1 | PDU1 (unclustered) |
| 1203605 | Actinobacteria | *Propionibacterium* sp. oral taxon 192 str. F0372 | 1 | 0 | 0 | 5 | 2 | 2 | PDU/EUT |
| 675635 | Actinobacteria | *Pseudonocardia dioxanivorans* CB1190 | 1 | 0 | 0 | 2 | 1 | 1 | MIC1 |
| 101510 | Actinobacteria | *Rhodococcus jostii* RHA1 | 1 | 0 | 0 | 2 | 3 | 2 | RMM1 |
| 263358 | Actinobacteria | *Verrucosispora maris* AB-18-032 | 1 | 0 | 0 | 2 | 0 | 1 | MIC (unclustered) |
|  |  |  |  |  |  |  |  |  |  |
| 517418 | Chlorobi | *Chloroherpeton thalassium* ATCC 35110 | 3 | 0 | 3 | 2 | 0 | 3 | all satellite-like |
|  |  |  |  |  |  |  |  |  |  |
| 926569 | Chloroflexi | *Anaerolinea thermophila* UNI-1 | 1 | 0 | 0 | 2 | 2 | 1 | EUT1 |
|  |  |  |  |  |  |  |  |  |  |
| 329726 | Cyanobacteria | *Acaryochloris marina* MBIC11017 | 5 | 4 | 0 | 6 | 2 | 1 | Beta-Carboxysome |
| 272123 | Cyanobacteria | *Anabaena cylindrica* PCC 7122 | 4 | 3 | 0 | 5 | 2 | 1 | Beta-Carboxysome |
| 46234 | Cyanobacteria | *Anabaena* sp. 90 | 3 | 2 | 0 | 4 | 2 | 1 | Beta-Carboxysome |
| 240292 | Cyanobacteria | *Anabaena variabilis* ATCC 29413 | 3 | 2 | 0 | 4 | 2 | 1 | Beta-Carboxysome |
| 696747 | Cyanobacteria | *Arthrospira platensis* NIES-39 | 3 | 2 | 0 | 4 | 2 | 1 | Beta-Carboxysome |
| 1170562 | Cyanobacteria | *Calothrix* sp. PCC 6303 | 3 | 2 | 0 | 4 | 2 | 1 | Beta-Carboxysome |
| 99598 | Cyanobacteria | *Calothrix* sp. PCC 7507 | 3 | 2 | 0 | 4 | 2 | 1 | Beta-Carboxysome |
| 1173020 | Cyanobacteria | *Chamaesiphon minutus* PCC 6605 | 2 | 1 | 0 | 4 | 2 | 1 | Beta-Carboxysome |
| 251229 | Cyanobacteria | *Chroococcidiopsis thermalis* PCC 7203 | 4 | 3 | 0 | 5 | 2 | 1 | Beta-Carboxysome |
| 1173022 | Cyanobacteria | *Crinalium epipsammum* PCC 9333 | 3 | 2 | 0 | 3 | 2 | 1 | Beta-Carboxysome |
| 755178 | Cyanobacteria | *Cyanobacterium aponinum* PCC 10605 | 4 | 3 | 0 | 4 | 2 | 1 | Beta-Carboxysome |
| 292563 | Cyanobacteria | *Cyanobacterium stanieri* PCC 7202 | 4 | 3 | 0 | 5 | 2 | 1 | Beta-Carboxysome |
| 43989 | Cyanobacteria | *Cyanothece* sp. ATCC 51142 | 4 | 3 | 0 | 4 | 2 | 1 | Beta-Carboxysome |
| 65393 | Cyanobacteria | *Cyanothece* sp. PCC 7424 | 4 | 3 | 0 | 5 | 2 | 1 | Beta-Carboxysome |
| 395961 | Cyanobacteria | *Cyanothece* sp. PCC 7425 | 5 | 4 | 0 | 5 | 2 | 1 | Beta-Carboxysome |
| 497965 | Cyanobacteria | *Cyanothece* sp. PCC 7822 | 3 | 2 | 0 | 5 | 1 | 1 | Beta-Carboxysome |
| 41431 | Cyanobacteria | *Cyanothece* sp. PCC 8801 | 4 | 3 | 0 | 4 | 2 | 1 | Beta-Carboxysome |
| 56107 | Cyanobacteria | *Cylindrospermum stagnale* PCC 7417 | 3 | 2 | 0 | 4 | 2 | 1 | Beta-Carboxysome |
| 13035 | Cyanobacteria | *Dactylococcopsis salina* PCC 8305 | 4 | 3 | 0 | 4 | 2 | 1 | Beta-Carboxysome |
| 359140 | Cyanobacteria | environmental samples uncultured marine type-A *Synechococcus* 5B2 | 1 | 0 | 0 | 2 | 1 | 2 | Alpha-Carboxysome |
| 364149 | Cyanobacteria | environmental samples uncultured marine type-A *Synechococcus* GOM 3M9 | 1 | 0 | 0 | 2 | 1 | 2 | Alpha-Carboxysome |
| 1173025 | Cyanobacteria | *Geitlerinema* sp. PCC 7407 | 3 | 2 | 0 | 4 | 2 | 1 | Beta-Carboxysome |
| 251221 | Cyanobacteria | *Gloeobacter violaceus* PCC 7421 | 2 | 1 | 0 | 2 | 2 | 1 | Beta-Carboxysome |
| 1173026 | Cyanobacteria | *Gloeocapsa* sp. PCC 7428 | 3 | 2 | 0 | 3 | 2 | 1 | Beta-Carboxysome |
| 65093 | Cyanobacteria | *Halothece* sp. PCC 7418 | 4 | 3 | 0 | 4 | 2 | 1 | Beta-Carboxysome |
| 111781 | Cyanobacteria | *Leptolyngbya* sp. PCC 7376 | 4 | 3 | 0 | 4 | 2 | 1 | Beta-Carboxysome |
| 1173027 | Cyanobacteria | *Microcoleus* sp. PCC 7113 | 4 | 3 | 0 | 4 | 2 | 1 | Beta-Carboxysome |
| 449447 | Cyanobacteria | *Microcystis aeruginosa* NIES-843 | 4 | 3 | 0 | 4 | 2 | 1 | Beta-Carboxysome |
| 63737 | Cyanobacteria | *Nostoc punctiforme* PCC 73102 | 3 | 2 | 0 | 4 | 2 | 1 | Beta-Carboxysome |
| 317936 | Cyanobacteria | *Nostoc* sp. PCC 7107 | 3 | 2 | 0 | 4 | 2 | 1 | Beta-Carboxysome |
| 103690 | Cyanobacteria | *Nostoc* sp. PCC 7120 | 3 | 2 | 0 | 4 | 2 | 1 | Beta-Carboxysome |
| 28072 | Cyanobacteria | *Nostoc* sp. PCC 7524 | 4 | 3 | 0 | 5 | 2 | 1 | Beta-Carboxysome |
| 56110 | Cyanobacteria | *Oscillatoria acuminata* PCC 6304 | 2 | 0 | 1 | 4 | 2 | 1 | Beta-Carboxysome |
| 179408 | Cyanobacteria | *Oscillatoria nigro-viridis* PCC 7112 | 3 | 2 | 0 | 3 | 2 | 1 | Beta-Carboxysome |
| 118163 | Cyanobacteria | *Pleurocapsa* sp. PCC 7327 | 4 | 3 | 0 | 4 | 2 | 1 | Beta-Carboxysome |
| 146891 | Cyanobacteria | *Prochlorococcus marinus* str. AS9601 | 1 | 0 | 0 | 2 | 0 | 2 | Alpha-Carboxysome |
| 93059 | Cyanobacteria | *Prochlorococcus marinus* str. MIT 9211 | 1 | 0 | 0 | 2 | 1 | 2 | Alpha-Carboxysome |
| 93060 | Cyanobacteria | *Prochlorococcus marinus* str. MIT 9215 | 1 | 0 | 0 | 1 | 1 | 2 | Alpha-Carboxysome |
| 167546 | Cyanobacteria | *Prochlorococcus marinus* str. MIT 9301 | 1 | 0 | 0 | 2 | 0 | 2 | Alpha-Carboxysome |
| 59922 | Cyanobacteria | *Prochlorococcus marinus* str. MIT 9303 | 1 | 0 | 0 | 2 | 1 | 2 | Alpha-Carboxysome |
| 74546 | Cyanobacteria | *Prochlorococcus marinus* str. MIT 9312 | 1 | 0 | 0 | 1 | 1 | 2 | Alpha-Carboxysome |
| 74547 | Cyanobacteria | *Prochlorococcus marinus* str. MIT 9313 | 1 | 0 | 0 | 2 | 1 | 2 | Alpha-Carboxysome |
| 167542 | Cyanobacteria | *Prochlorococcus marinus* str. MIT 9515 | 1 | 0 | 0 | 1 | 1 | 2 | Alpha-Carboxysome |
| 167555 | Cyanobacteria | *Prochlorococcus marinus* str. NATL1A | 1 | 0 | 0 | 3 | 0 | 2 | Alpha-Carboxysome |
| 59920 | Cyanobacteria | *Prochlorococcus marinus* str. NATL2A | 1 | 0 | 0 | 3 | 0 | 2 | Alpha-Carboxysome |
| 167539 | Cyanobacteria | *Prochlorococcus marinus* subsp. *marinus* str. CCMP1375 | 1 | 0 | 0 | 2 | 1 | 2 | Alpha-Carboxysome |
| 59919 | Cyanobacteria | *Prochlorococcus marinus* subsp. *pastoris* str. CCMP1986 | 1 | 0 | 0 | 1 | 1 | 2 | Alpha-Carboxysome |
| 82654 | Cyanobacteria | *Pseudanabaena* sp. PCC 7367 | 3 | 2 | 0 | 4 | 2 | 1 | Beta-Carboxysome |
| 373994 | Cyanobacteria | *Rivularia* sp. PCC 7116 | 3 | 2 | 0 | 4 | 2 | 1 | Beta-Carboxysome |
| 111780 | Cyanobacteria | *Stanieria cyanosphaera* PCC 7437 | 4 | 3 | 0 | 5 | 2 | 1 | Beta-Carboxysome |
| 269084 | Cyanobacteria | *Synechococcus elongatus* PCC 6301 | 3 | 2 | 0 | 3 | 2 | 1 | Beta-Carboxysome |
| 64471 | Cyanobacteria | *Synechococcus* sp. CC9311 | 1 | 0 | 0 | 2 | 1 | 2 | Alpha-Carboxysome |
| 110662 | Cyanobacteria | *Synechococcus* sp. CC9605 | 1 | 0 | 0 | 2 | 1 | 2 | Alpha-Carboxysome |
| 316279 | Cyanobacteria | *Synechococcus* sp. CC9902 | 1 | 0 | 0 | 2 | 1 | 2 | Alpha-Carboxysome |
| 321332 | Cyanobacteria | *Synechococcus* sp. JA-2-3B'a(2-13) | 2 | 1 | 0 | 2 | 2 | 1 | Beta-Carboxysome |
| 321327 | Cyanobacteria | *Synechococcus* sp. JA-3-3Ab | 2 | 1 | 0 | 2 | 2 | 1 | Beta-Carboxysome |
| 195253 | Cyanobacteria | *Synechococcus* sp. PCC 6312 | 5 | 4 | 0 | 4 | 2 | 1 | Beta-Carboxysome |
| 32049 | Cyanobacteria | *Synechococcus* sp. PCC 7002 | 4 | 3 | 0 | 4 | 2 | 1 | Beta-Carboxysome |
| 1173263 | Cyanobacteria | *Synechococcus* sp. PCC 7502 | 4 | 3 | 0 | 4 | 2 | 1 | Beta-Carboxysome |
| 316278 | Cyanobacteria | *Synechococcus* sp. RCC307 | 1 | 0 | 0 | 2 | 1 | 2 | Alpha-Carboxysome |
| 32051 | Cyanobacteria | *Synechococcus* sp. WH 7803 | 1 | 0 | 0 | 2 | 1 | 2 | Alpha-Carboxysome |
| 84588 | Cyanobacteria | *Synechococcus* sp. WH 8102 | 1 | 0 | 0 | 2 | 1 | 2 | Alpha-Carboxysome |
| 1148 | Cyanobacteria | *Synechocystis* sp. PCC 6803 | 4 | 3 | 0 | 4 | 2 | 1 | Beta-Carboxysome |
| 197221 | Cyanobacteria | *Thermosynechococcus elongatus* BP-1 | 4 | 3 | 0 | 4 | 2 | 1 | Beta-Carboxysome |
| 203124 | Cyanobacteria | *Trichodesmium erythraeum* IMS101 | 3 | 2 | 0 | 4 | 2 | 1 | Beta-Carboxysome |
| 551115 | Cyanobacteria | *Trichormus* 'Nostoc azollae' 0708 | 4 | 3 | 0 | 5 | 2 | 1 | Beta-Carboxysome |
|  |  |  |  |  |  |  |  |  |  |
| 931626 | Firmicutes | *Acetobacterium woodii* DSM 1030 | 4 | 1 | 2 | 4 | 3 | 1 | PDU1 (unclustered) |
| 293826 | Firmicutes | *Alkaliphilus metalliredigens* QYMF | 3 | 0 | 0 | 12 | 3 | 3 | BUF, EUT2B, GRM1 |
| 350688 | Firmicutes | *Alkaliphilus oremlandii* OhILAs | 3 | 0 | 0 | 12 | 2 | 3 | BUF, EUT2 (unclustered), GRM1 |
| 592022 | Firmicutes | *Bacillus megaterium* DSM 319 | 1 | 0 | 0 | 3 | 1 | 1 | EUT2C |
| 545693 | Firmicutes | *Bacillus megaterium* QM B1551 | 1 | 0 | 0 | 3 | 1 | 1 | EUT2C |
| 439292 | Firmicutes | *Bacillus selenitireducens* MLS10 | 1 | 0 | 0 | 5 | 0 | 1 | PVM-like |
| 1204343 | Firmicutes | *Bacillus subtilis* BEST7613 | 4 | 3 | 0 | 4 | 2 | 1 | Beta-Carboxysome (synthetic) |
| 358681 | Firmicutes | *Brevibacillus brevis* NBRC 100599 | 1 | 0 | 0 | 3 | 1 | 1 | EUT2 (unclustered) |
| 246194 | Firmicutes | *Carboxydothermus hydrogenoformans* Z-2901 | 1 | 0 | 0 | 3 | 1 | 1 | BUF |
| 1234679 | Firmicutes | *Carnobacterium maltaromaticum* LMA28 | 1 | 0 | 0 | 3 | 1 | 1 | EUT2A |
| 1128398 | Firmicutes | *Clostridium acidurici* 9a | 3 | 2 | 0 | 4 | 1 | 1 | BUF |
| 290402 | Firmicutes | *Clostridium beijerinckii* NCIMB 8052 | 1 | 0 | 0 | 4 | 2 | 1 | GRM3 |
| 413999 | Firmicutes | *Clostridium botulinum* A str. ATCC 3502 | 1 | 0 | 0 | 6 | 0 | 1 | GRM1 |
| 498214 | Firmicutes | *Clostridium botulinum* A3 str. Loch Maree | 2 | 1 | 0 | 6 | 1 | 1 | GRM1 |
| 508765 | Firmicutes | *Clostridium botulinum* B str. Eklund 17B | 2 | 0 | 0 | 8 | 3 | 2 | GRM1, MUF |
| 498213 | Firmicutes | *Clostridium botulinum* B1 str. Okra | 2 | 1 | 0 | 6 | 1 | 1 | GRM1 |
| 515621 | Firmicutes | *Clostridium botulinum* Ba4 str. 657 | 1 | 0 | 0 | 5 | 0 | 1 | GRM1 |
| 929506 | Firmicutes | *Clostridium botulinum* BKT015925 | 1 | 0 | 0 | 4 | 0 | 1 | GRM1 |
| 508767 | Firmicutes | *Clostridium botulinum* E3 str. Alaska E43 | 2 | 0 | 0 | 9 | 3 | 2 | GRM1, MUF |
| 441772 | Firmicutes | *Clostridium botulinum* F str. Langeland | 2 | 1 | 0 | 6 | 1 | 1 | GRM1 |
| 941968 | Firmicutes | *Clostridium botulinum* H04402 065 | 1 | 0 | 0 | 6 | 0 | 1 | GRM1 |
| 717608 | Firmicutes | *Clostridium* cf. *saccharolyticum* K10 | 1 | 0 | 0 | 4 | 2 | 1 | GRM3 |
| 272563 | Firmicutes | *Clostridium difficile* 630 | 1 | 0 | 0 | 3 | 2 | 1 | EUT2A |
| 645462 | Firmicutes | *Clostridium difficile* CD196 | 1 | 0 | 0 | 3 | 2 | 1 | EUT2A |
| 431943 | Firmicutes | *Clostridium kluyveri* DSM 555 | 4 | 1 | 1 | 5 | 5 | 1 | ETU, PDU-like/PDU3 |
| 583346 | Firmicutes | *Clostridium kluyveri* NBRC 12016 | 4 | 1 | 1 | 5 | 5 | 1 | ETU, PDU-like/PDU3 |
| 642492 | Firmicutes | *Clostridium lentocellum* DSM 5427 | 1 | 0 | 0 | 3 | 2 | 1 | EUT2 (unclustered) |
| 748727 | Firmicutes | *Clostridium ljungdahlii* DSM 13528 | 5 | 2 | 1 | 11 | 3 | 3 | GRM3 |
| 386415 | Firmicutes | *Clostridium novyi* NT | 1 | 0 | 0 | 2 | 1 | 1 | GRM3 |
| 195103 | Firmicutes | *Clostridium perfringens* ATCC 13124 | 1 | 0 | 0 | 3 | 2 | 1 | EUT2A |
| 195102 | Firmicutes | *Clostridium perfringens* str. 13 | 1 | 0 | 0 | 3 | 2 | 1 | EUT2A |
| 357809 | Firmicutes | *Clostridium phytofermentans* ISDg | 3 | 0 | 0 | 14 | 3 | 3 | EUT2 (unclustered), GRM1, GRM5 |
| 610130 | Firmicutes | *Clostridium saccharolyticum* WM1 | 3 | 0 | 0 | 15 | 1 | 3 | EUT2 (unclustered), GRM1, PVM-like |
| 212717 | Firmicutes | *Clostridium tetani* E88 | 2 | 0 | 0 | 4 | 2 | 0 | EUT2A, GRM1 |
| 717962 | Firmicutes | *Coprococcus catus* GD/7 | 1 | 0 | 0 | 2 | 2 | 1 | EUT (unclustered) |
| 756499 | Firmicutes | *Desulfitobacterium dehalogenans* ATCC 51507 | 2 | 0 | 0 | 8 | 2 | 2 | GRM1 |
| 272564 | Firmicutes | *Desulfitobacterium hafniense* DCB-2 | 3 | 0 | 0 | 8 | 3 | 3 | EUT3, GRM1 |
| 138119 | Firmicutes | *Desulfitobacterium hafniense* Y51 | 3 | 0 | 0 | 8 | 3 | 3 | EUT3, GRM1 |
| 646529 | Firmicutes | *Desulfosporosinus acidiphilus* SJ4 | 1 | 0 | 0 | 6 | 1 | 1 | GRM1 |
| 768704 | Firmicutes | *Desulfosporosinus meridiei* DSM 13257 | 3 | 0 | 0 | 10 | 5 | 3 | EUT2B, EUT2D, GRM3 |
| 768706 | Firmicutes | *Desulfosporosinus orientis* DSM 765 | 3 | 0 | 0 | 11 | 5 | 3 | EUT (unclustered), EUT2B, GRM3 |
| 349161 | Firmicutes | *Desulfotomaculum reducens* MI-1 | 2 | 0 | 0 | 10 | 3 | 2 | EUT2B, GRM1 |
| 696281 | Firmicutes | *Desulfotomaculum ruminis* DSM 2154 | 1 | 0 | 0 | 6 | 1 | 1 | GRM1 |
| 936153 | Firmicutes | *Enterococcus faecalis* 62 | 1 | 0 | 0 | 3 | 1 | 1 | EUT2A |
| 474186 | Firmicutes | *Enterococcus faecalis* OG1RF | 1 | 0 | 0 | 3 | 1 | 1 | EUT2A |
| 226185 | Firmicutes | *Enterococcus faecalis* V583 | 1 | 0 | 0 | 3 | 1 | 1 | EUT2A |
| 903814 | Firmicutes | *Eubacterium limosum* KIST612 | 4 | 1 | 2 | 4 | 3 | 1 | PDU1 (unclustered) |
| 581103 | Firmicutes | *Geobacillus* sp. Y4.1MC1 | 1 | 0 | 0 | 3 | 1 | 1 | PDU1 (unclustered) |
| 656519 | Firmicutes | *Halanaerobium hydrogeniformans* | 2 | 0 | 0 | 5 | 4 | 1 | EUT (unclustered), PDU1 (unclustered) |
| 572479 | Firmicutes | *Halanaerobium praevalens* DSM 2228 | 1 | 0 | 0 | 4 | 3 | 1 | EUT2B |
| 387344 | Firmicutes | *Lactobacillus brevis* ATCC 367 | 2 | 1 | 0 | 4 | 1 | 2 | PDU1 (unclustered) |
| 1001583 | Firmicutes | *Lactobacillus brevis* KB290 | 2 | 1 | 0 | 4 | 1 | 2 | PDU1 (unclustered) |
| 1033837 | Firmicutes | *Lactobacillus kefiranofaciens* ZW3 | 1 | 0 | 1 | 1 | 0 | 0 | all satellite-like |
| 557436 | Firmicutes | *Lactobacillus reuteri* DSM 20016 | 1 | 0 | 0 | 4 | 1 | 1 | PDU1C |
| 557433 | Firmicutes | *Lactobacillus reuteri* JCM 1112 | 1 | 0 | 0 | 4 | 1 | 1 | PDU1C |
| 491077 | Firmicutes | *Lactobacillus reuteri* SD2112 | 1 | 0 | 0 | 4 | 1 | 1 | PDU1C |
| 272626 | Firmicutes | *Listeria innocua* Clip11262 | 1 | 0 | 0 | 6 | 4 | 2 | PDU/EUT |
| 881621 | Firmicutes | *Listeria ivanovii* subsp. *ivanovii* PAM 55 | 1 | 0 | 0 | 6 | 4 | 2 | PDU/EUT |
| 1639 | Firmicutes | *Listeria monocytogenes* | 1 | 0 | 0 | 6 | 4 | 2 | PDU/EUT |
| 653938 | Firmicutes | *Listeria monocytogenes* 08-5578 | 1 | 0 | 0 | 6 | 4 | 2 | PDU/EUT |
| 393133 | Firmicutes | *Listeria monocytogenes* 10403S | 1 | 0 | 0 | 6 | 4 | 2 | PDU/EUT |
| 169963 | Firmicutes | *Listeria monocytogenes* EGD-e | 1 | 0 | 0 | 6 | 4 | 2 | PDU/EUT |
| 393127 | Firmicutes | *Listeria monocytogenes* Finland 1998 | 1 | 0 | 0 | 6 | 4 | 2 | PDU/EUT |
| 552536 | Firmicutes | *Listeria monocytogenes* HCC23 | 1 | 0 | 0 | 6 | 4 | 2 | PDU/EUT |
| 393130 | Firmicutes | *Listeria monocytogenes* J0161 | 1 | 0 | 0 | 6 | 4 | 2 | PDU/EUT |
| 563174 | Firmicutes | *Listeria monocytogenes* L99 | 1 | 0 | 0 | 6 | 4 | 2 | PDU/EUT |
| 1234142 | Firmicutes | *Listeria monocytogenes* N53-1 | 1 | 0 | 0 | 6 | 4 | 3 | PDU/EUT |
| 568819 | Firmicutes | *Listeria monocytogenes* serotype 4b str. CLIP 80459 | 1 | 0 | 0 | 6 | 4 | 2 | PDU/EUT |
| 265669 | Firmicutes | *Listeria monocytogenes* serotype 4b str. F2365 | 1 | 0 | 0 | 6 | 4 | 2 | PDU/EUT |
| 882097 | Firmicutes | *Listeria monocytogenes* SLCC2376 | 1 | 0 | 0 | 6 | 4 | 2 | PDU/EUT |
| 879088 | Firmicutes | *Listeria monocytogenes* SLCC2378 | 1 | 0 | 0 | 7 | 4 | 2 | PDU/EUT |
| 932919 | Firmicutes | *Listeria monocytogenes* SLCC2755 | 1 | 0 | 0 | 6 | 4 | 2 | PDU/EUT |
| 683837 | Firmicutes | *Listeria seeligeri* serovar 1/2b str. SLCC3954 | 1 | 0 | 0 | 6 | 4 | 2 | PDU/EUT |
| 386043 | Firmicutes | *Listeria welshimeri* serovar 6b str. SLCC5334 | 1 | 0 | 0 | 6 | 4 | 2 | PDU/EUT |
| 444177 | Firmicutes | *Lysinibacillus sphaericus* C3-41 | 1 | 0 | 0 | 3 | 1 | 1 | EUT2 (unclustered) |
| 697281 | Firmicutes | *Mahella australiensis* 50-1 BON | 1 | 0 | 0 | 4 | 1 | 1 | MIC (unclustered) |
| 693746 | Firmicutes | *Oscillibacter valericigenes* Sjm18-20 | 1 | 0 | 0 | 4 | 2 | 1 | GRM3 |
| 701521 | Firmicutes | *Pediococcus claussenii* ATCC BAA-344 | 1 | 0 | 0 | 4 | 1 | 1 | PDU1 (unclustered) |
| 1511 | Firmicutes | *Peptostreptococcaceae* *sticklandii* | 2 | 0 | 0 | 7 | 2 | 2 | EUT2B, PVM-like |
| 622312 | Firmicutes | *Roseburia inulinivorans* DSM 16841 | 1 | 0 | 0 | 5 | 1 | 1 | GRM5 |
| 657314 | Firmicutes | *Ruminococcus obeum* A2-162 | 4 | 2 | 1 | 8 | 2 | 1 | GRM5 |
| 657323 | Firmicutes | *Ruminococcus* sp. SR1/5 | 4 | 2 | 1 | 8 | 2 | 1 | GRM5 |
| 657313 | Firmicutes | *Ruminococcus torques* L2-14 | 4 | 2 | 1 | 8 | 2 | 1 | GRM5 |
| 663954 | Firmicutes | *Streptococcus dysgalactiae* subsp. *equisimilis* ATCC 12394 | 1 | 0 | 0 | 7 | 0 | 1 | GRM1 |
| 617121 | Firmicutes | *Streptococcus dysgalactiae* subsp. *equisimilis* RE378 | 1 | 0 | 0 | 5 | 0 | 1 | GRM1 |
| 1318633 | Firmicutes | *Streptococcus iniae* SF1 | 1 | 0 | 0 | 6 | 1 | 1 | GRM1 |
| 1316583 | Firmicutes | *Streptococcus intermedius* ATCC 27335 | 1 | 0 | 0 | 7 | 0 | 1 | GRM1 |
| 591365 | Firmicutes | *Streptococcus intermedius* JTH08 | 1 | 0 | 0 | 4 | 0 | 1 | GRM1 |
| 388919 | Firmicutes | *Streptococcus sanguinis* SK36 | 1 | 0 | 0 | 4 | 2 | 1 | PDU/EUT |
| 292459 | Firmicutes | *Symbiobacterium thermophilum* IAM 14863 | 1 | 0 | 0 | 1 | 0 | 0 | EUT (unclustered) |
| 1209989 | Firmicutes | *Tepidanaerobacter acetatoxydans* Re1 | 1 | 0 | 1 | 1 | 0 | 0 | all satellite-like |
| 1089553 | Firmicutes | *Thermacetogenium phaeum* DSM 12270 | 1 | 0 | 0 | 3 | 2 | 1 | EUT2B |
| 635013 | Firmicutes | *Thermincola potens* JR | 2 | 0 | 0 | 7 | 4 | 2 | EUT2D, PDU1 (unclustered) |
| 573062 | Firmicutes | *Thermoanaerobacter* sp. X513 | 1 | 0 | 0 | 3 | 2 | 1 | PDU1D |
| 399726 | Firmicutes | *Thermoanaerobacter* sp. X514 | 1 | 0 | 0 | 3 | 2 | 1 | PDU1D |
| 1094508 | Firmicutes | *Thermoanaerobacterium saccharolyticum* JW/SL-YS485 | 1 | 0 | 0 | 4 | 1 | 1 | PDU1 (unclustered) |
| 698948 | Firmicutes | *Thermoanaerobacterium thermosaccharolyticum* M0795 | 1 | 0 | 0 | 3 | 1 | 1 | PDU1 (unclustered) |
| 858215 | Firmicutes | *Thermoanaerobacterium xylanolyticum* LX-11 | 1 | 0 | 0 | 4 | 1 | 1 | PDU1 (unclustered) |
| 555079 | Firmicutes | *Thermosediminibacter oceani* DSM 16646 | 1 | 0 | 0 | 2 | 2 | 1 | PDU1 (unclustered) |
|  |  |  |  |  |  |  |  |  |  |
| 469607 | Fusobacteria | *Fusobacterium nucleatum* subsp. *animalis* 4_8 | 2 | 0 | 0 | 5 | 2 | 2 | EUT2A, PDU1 (unclustered) |
| 190304 | Fusobacteria | *Fusobacterium nucleatum* subsp. *nucleatum* ATCC 25586 | 1 | 0 | 0 | 3 | 1 | 1 | EUT2A |
| 572544 | Fusobacteria | *Ilyobacter polytropus* DSM 2926 | 1 | 0 | 0 | 2 | 3 | 1 | PDU1 (unclustered) |
| 523794 | Fusobacteria | *Leptotrichia buccalis* C-1013-b | 1 | 0 | 0 | 5 | 1 | 1 | EUT2A |
| 526218 | Fusobacteria | *Sebaldella termitidis* ATCC 33386 | 2 | 1 | 0 | 5 | 3 | 1 | PDU/EUT |
|  |  |  |  |  |  |  |  |  |  |
| 945713 | Ignavibacteriae | *Ignavibacterium album* JCM 16511 | 1 | 0 | 1 | 2 | 0 | 0 | all satellite-like |
| 1191523 | Ignavibacteriae | *Melioribacter roseus* P3M-2 | 5 | 3 | 1 | 5 | 1 | 7 | PVM-like |
|  |  |  |  |  |  |  |  |  |  |
| 575540 | Planctomycetes | *Isosphaera* *pallida* ATCC 43644 | 1 | 0 | 0 | 3 | 0 | 3 | PVM |
| 530564 | Planctomycetes | *Pirellula staleyi* DSM 6068 | 2 | 1 | 0 | 2 | 1 | 3 | PVM |
| 756272 | Planctomycetes | *Planctomyces brasiliensis* DSM 5305 | 1 | 0 | 0 | 2 | 0 | 3 | PVM |
| 521674 | Planctomycetes | *Planctomyces limnophilus* DSM 3776 | 1 | 0 | 0 | 2 | 0 | 3 | PVM |
| 243090 | Planctomycetes | *Rhodopirellula baltica* SH 1 | 1 | 0 | 0 | 2 | 0 | 3 | PVM |
| 886293 | Planctomycetes | *Singulisphaera acidiphila* DSM 18658 | 1 | 0 | 0 | 3 | 0 | 3 | PVM |
|  |  |  |  |  |  |  |  |  |  |
| 288000 | Alphaproteobacteria | *Bradyrhizobium* sp. BTAi1 | 1 | 0 | 0 | 2 | 0 | 2 | Alpha-Carboxysome |
| 114615 | Alphaproteobacteria | *Bradyrhizobium* sp. ORS 278 | 1 | 0 | 0 | 2 | 0 | 2 | Alpha-Carboxysome |
| 323098 | Alphaproteobacteria | *Nitrobacter winogradskyi* Nb-255 | 1 | 0 | 0 | 2 | 1 | 2 | Alpha-Carboxysome |
| 272942 | Alphaproteobacteria | *Rhodobacter capsulatus* SB 1003 | 1 | 0 | 0 | 3 | 1 | 1 | GRM3 |
| 316056 | Alphaproteobacteria | *Rhodopseudomonas palustris* BisB18 | 1 | 0 | 0 | 3 | 1 | 1 | GRM3 |
| 269796 | Alphaproteobacteria | *Rhodospirillum rubrum* ATCC 11170 | 1 | 0 | 0 | 4 | 1 | 1 | GRM3 |
| 1036743 | Alphaproteobacteria | *Rhodospirillum rubrum* F11 | 1 | 0 | 0 | 4 | 1 | 1 | GRM3 |
|  |  |  |  |  |  |  |  |  |  |
| 420662 | Betaproteobacteria | *Methylibium petroleiphilum* PM1 | 1 | 0 | 0 | 2 | 1 | 1 | MIC1 |
| 335283 | Betaproteobacteria | *Nitrosomonas eutropha* C91 | 1 | 0 | 0 | 2 | 1 | 2 | Alpha-Carboxysome |
| 292415 | Betaproteobacteria | *Thiobacillus denitrificans* ATCC 25259 | 1 | 0 | 0 | 3 | 0 | 2 | Alpha-Carboxysome |
| 75379 | Betaproteobacteria | *Thiomonas intermedia* K12 | 1 | 0 | 0 | 4 | 0 | 2 | Alpha-Carboxysome |
| 426114 | Betaproteobacteria | *Thiomonas* sp. 3As | 1 | 0 | 0 | 4 | 0 | 2 | Alpha-Carboxysome |
| 391735 | Betaproteobacteria | *Verminephrobacter eiseniae* EF01-2 | 1 | 0 | 0 | 1 | 2 | 1 | RMM-like |
|  |  |  |  |  |  |  |  |  |  |
| 990288 | Gammaproteobacteria | *Acidithiobacillus caldus* SM-1 | 1 | 0 | 0 | 2 | 1 | 2 | Alpha-Carboxysome |
| 743299 | Gammaproteobacteria | *Acidithiobacillus ferrivorans* SS3 | 1 | 0 | 0 | 2 | 0 | 2 | Alpha-Carboxysome |
| 243159 | Gammaproteobacteria | *Acidithiobacillus ferrooxidans* ATCC 23270 | 1 | 0 | 0 | 3 | 0 | 2 | Alpha-Carboxysome |
| 380394 | Gammaproteobacteria | *Acidithiobacillus ferrooxidans* ATCC 53993 | 1 | 0 | 0 | 3 | 0 | 2 | Alpha-Carboxysome |
| 1288394 | Gammaproteobacteria | *Aeromonas hydrophila* ML09-119 | 1 | 0 | 0 | 4 | 0 | 1 | GRM2 |
| 380703 | Gammaproteobacteria | *Aeromonas hydrophila* subsp. *hydrophila* ATCC 7966 | 1 | 0 | 0 | 4 | 0 | 1 | GRM2 |
| 572477 | Gammaproteobacteria | *Allochromatium vinosum* DSM 180 | 2 | 1 | 0 | 2 | 1 | 2 | Alpha-Carboxysome |
| 1261127 | Gammaproteobacteria | *Citrobacter amalonaticus* Y19 | 1 | 0 | 0 | 4 | 2 | 1 | PDU1A |
| 290338 | Gammaproteobacteria | *Citrobacter koseri* ATCC BAA-895 | 2 | 0 | 0 | 7 | 3 | 2 | EUT1, PDU1A |
| 637910 | Gammaproteobacteria | *Citrobacter rodentium* ICC168 | 2 | 0 | 0 | 7 | 3 | 2 | EUT1, PDU1A |
| 1028307 | Gammaproteobacteria | *Enterobacter aerogenes* KCTC 2190 | 1 | 0 | 0 | 4 | 0 | 1 | GRM2 |
| 701347 | Gammaproteobacteria | *Enterobacter lignolyticus* SCF1 | 2 | 0 | 0 | 7 | 3 | 2 | EUT1, PDU1A |
| 469613 | Gammaproteobacteria | *Enterobacteriaceae bacterium* 9_2_54FAA | 1 | 0 | 0 | 4 | 2 | 1 | PDU1 (unclustered) |
| 693444 | Gammaproteobacteria | *Enterobacteriaceae bacterium* strain FGI 57 | 1 | 0 | 0 | 3 | 1 | 1 | EUT1 |
| 216592 | Gammaproteobacteria | *Escherichia coli* 042 | 1 | 0 | 0 | 3 | 1 | 1 | EUT1 |
| 362663 | Gammaproteobacteria | *Escherichia coli* 536 | 2 | 0 | 0 | 7 | 1 | 2 | EUT1, GRM2 |
| 655817 | Gammaproteobacteria | *Escherichia coli* ABU 83972 | 1 | 0 | 0 | 3 | 1 | 1 | EUT1 |
| 405955 | Gammaproteobacteria | *Escherichia coli* APEC O1 | 2 | 0 | 0 | 5 | 2 | 2 | EUT1, GRM3 |
| 469008 | Gammaproteobacteria | *Escherichia coli* BL21(DE3) | 1 | 0 | 0 | 3 | 1 | 1 | EUT1 |
| 199310 | Gammaproteobacteria | *Escherichia coli* CFT073 | 2 | 0 | 0 | 6 | 2 | 2 | EUT1, GRM3 |
| 331111 | Gammaproteobacteria | *Escherichia coli* E24377A | 2 | 0 | 0 | 7 | 3 | 2 | EUT1, PDU1B |
| 585397 | Gammaproteobacteria | *Escherichia coli* ED1a | 2 | 0 | 0 | 6 | 2 | 2 | EUT1, GRM3 |
| 585057 | Gammaproteobacteria | *Escherichia coli* IAI39 | 2 | 0 | 0 | 7 | 1 | 2 | EUT1, GRM2 |
| 595495 | Gammaproteobacteria | *Escherichia coli* KO11FL | 1 | 0 | 0 | 3 | 1 | 1 | EUT1 |
| 591946 | Gammaproteobacteria | *Escherichia coli* LF82 | 2 | 0 | 0 | 7 | 3 | 2 | EUT1, PDU1B |
| 574521 | Gammaproteobacteria | *Escherichia coli* O127:H6 str. E2348/69 | 3 | 1 | 0 | 7 | 3 | 2 | EUT1, PDU1B |
| 155864 | Gammaproteobacteria | *Escherichia coli* O157:H7 str. EDL933 | 1 | 0 | 0 | 3 | 1 | 1 | EUT1 |
| 386585 | Gammaproteobacteria | *Escherichia coli* O157:H7 str. Sakai | 1 | 0 | 0 | 3 | 1 | 1 | EUT1 |
| 1072459 | Gammaproteobacteria | *Escherichia coli* O7:K1 str. CE10 | 2 | 0 | 0 | 7 | 1 | 2 | EUT1, GRM2 |
| 585035 | Gammaproteobacteria | *Escherichia coli* S88 | 2 | 0 | 0 | 5 | 2 | 2 | EUT1, GRM3 |
| 409438 | Gammaproteobacteria | *Escherichia coli* SE11 | 1 | 0 | 0 | 3 | 1 | 1 | EUT1 |
| 439855 | Gammaproteobacteria | *Escherichia coli* SMS-3-5 | 1 | 0 | 0 | 3 | 1 | 1 | EUT1 |
| 511145 | Gammaproteobacteria | *Escherichia coli* str. K-12 substr. MG1655 | 1 | 0 | 0 | 3 | 1 | 1 | EUT1 |
| 585056 | Gammaproteobacteria | *Escherichia coli* UMN026 | 2 | 0 | 0 | 6 | 3 | 2 | EUT1, PDU1B |
| 696406 | Gammaproteobacteria | *Escherichia coli* UMNK88 | 1 | 0 | 0 | 3 | 1 | 1 | EUT1 |
| 364106 | Gammaproteobacteria | *Escherichia coli* UTI89 | 2 | 0 | 0 | 7 | 1 | 2 | EUT1, GRM2 |
| 585054 | Gammaproteobacteria | *Escherichia fergusonii* ATCC 35469 | 2 | 0 | 0 | 10 | 3 | 3 | EUT1, PDU/GRM |
| 555778 | Gammaproteobacteria | *Halothiobacillus neapolitanus* c2 | 1 | 0 | 0 | 3 | 1 | 2 | Alpha-Carboxysome |
| 1191061 | Gammaproteobacteria | *Klebsiella oxytoca* E718 | 3 | 0 | 0 | 11 | 3 | 3 | EUT1, GRM2, PDU1A |
| 1006551 | Gammaproteobacteria | *Klebsiella oxytoca* KCTC 1686 | 3 | 0 | 0 | 11 | 3 | 3 | EUT1, GRM2, PDU1A |
| 507522 | Gammaproteobacteria | *Klebsiella pneumoniae* 342 | 3 | 0 | 0 | 11 | 3 | 3 | EUT1, GRM2, PDU1A |
| 1049565 | Gammaproteobacteria | *Klebsiella pneumoniae* KCTC 2242 | 2 | 0 | 0 | 7 | 3 | 2 | EUT1, PDU1A |
| 1125630 | Gammaproteobacteria | *Klebsiella pneumoniae* subsp. *pneumoniae* HS11286 | 2 | 0 | 0 | 7 | 3 | 2 | EUT1, PDU1A |
| 272620 | Gammaproteobacteria | *Klebsiella pneumoniae* subsp. *pneumoniae* MGH 78578 | 2 | 0 | 0 | 7 | 3 | 2 | EUT1, PDU1A |
| 484021 | Gammaproteobacteria | *Klebsiella pneumoniae* subsp. *pneumoniae* NTUH-K2044 | 2 | 0 | 0 | 7 | 3 | 2 | EUT1, PDU1A |
| 1284812 | Gammaproteobacteria | *Klebsiella pneumoniae* UHKPC81 | 1 | 0 | 0 | 3 | 1 | 1 | EUT1 |
| 640131 | Gammaproteobacteria | *Klebsiella variicola* At-22 | 3 | 0 | 0 | 11 | 3 | 3 | EUT1, GRM2, PDU1A |
| 351348 | Gammaproteobacteria | *Marinobacter aquaeolei* VT8 | 1 | 0 | 0 | 3 | 2 | 1 | EUT1 |
| 1124991 | Gammaproteobacteria | *Morganella morganii* subsp. *morganii* KT | 1 | 0 | 0 | 3 | 2 | 1 | EUT1 |
| 698738 | Gammaproteobacteria | *Oleispira antarctica* RB-8 | 1 | 0 | 0 | 3 | 2 | 1 | EUT2B |
| 561231 | Gammaproteobacteria | *Pectobacterium wasabiae* WPP163 | 1 | 0 | 0 | 3 | 1 | 1 | GRM3 |
| 529507 | Gammaproteobacteria | *Proteus mirabilis* HI4320 | 1 | 0 | 0 | 4 | 0 | 1 | GRM2 |
| 1286170 | Gammaproteobacteria | *Raoultella ornithinolytica* B6 | 2 | 0 | 0 | 6 | 1 | 2 | GRM2, GRM3 |
| 41514 | Gammaproteobacteria | *Salmonella enterica* subsp. *arizonae* serovar 62:z4,z23:- | 2 | 0 | 0 | 7 | 3 | 2 | EUT1, PDU1A |
| 882884 | Gammaproteobacteria | *Salmonella enterica* subsp. *arizonae* serovar 62:z4,z23:- str. RSK2980 | 2 | 0 | 0 | 7 | 3 | 2 | EUT1, PDU1A |
| 454166 | Gammaproteobacteria | *Salmonella enterica* subsp. *enterica* serovar Agona str. SL483 | 2 | 0 | 0 | 6 | 3 | 2 | EUT1, PDU1A |
| 321314 | Gammaproteobacteria | *Salmonella enterica* subsp. *enterica* serovar Choleraesuis str. SC-B67 | 2 | 0 | 0 | 7 | 2 | 2 | EUT1, PDU1A |
| 439851 | Gammaproteobacteria | *Salmonella enterica* subsp. *enterica* serovar Dublin str. CT_02021853 | 2 | 0 | 0 | 6 | 3 | 1 | EUT1, PDU1A |
| 550537 | Gammaproteobacteria | *Salmonella enterica* subsp. *enterica* serovar Enteritidis str. P125109 | 2 | 0 | 0 | 7 | 3 | 2 | EUT1, PDU1A |
| 550538 | Gammaproteobacteria | *Salmonella enterica* subsp. *enterica* serovar Gallinarum str. 287/91 | 2 | 0 | 0 | 7 | 3 | 2 | EUT1, PDU1A |
| 1081093 | Gammaproteobacteria | *Salmonella enterica* subsp. *enterica* serovar Gallinarum/pullorum | 2 | 0 | 0 | 7 | 3 | 1 | EUT1, PDU1A |
| 454169 | Gammaproteobacteria | *Salmonella enterica* subsp. *enterica* serovar Heidelberg str. SL476 | 2 | 0 | 0 | 7 | 3 | 2 | EUT1, PDU1A |
| 1267753 | Gammaproteobacteria | *Salmonella enterica* subsp. *enterica* serovar Javiana str. CFSAN001992 | 2 | 0 | 0 | 7 | 3 | 2 | EUT1, PDU1A |
| 423368 | Gammaproteobacteria | *Salmonella enterica* subsp. *enterica* serovar Newport str. SL254 | 2 | 0 | 0 | 7 | 3 | 2 | EUT1, PDU1A |
| 295319 | Gammaproteobacteria | *Salmonella enterica* subsp. *enterica* serovar Paratyphi A str. ATCC 9150 | 2 | 0 | 0 | 7 | 3 | 2 | EUT1, PDU1A |
| 1016998 | Gammaproteobacteria | *Salmonella enterica* subsp. *enterica* serovar Paratyphi B str. SPB7 | 2 | 0 | 0 | 7 | 3 | 2 | EUT1, PDU1A |
| 476213 | Gammaproteobacteria | *Salmonella enterica* subsp. *enterica* serovar Paratyphi C strain RKS4594 | 2 | 0 | 0 | 6 | 3 | 1 | EUT1, PDU1A |
| 439843 | Gammaproteobacteria | *Salmonella enterica* subsp. *enterica* serovar Schwarzengrund str. CVM19633 | 2 | 0 | 0 | 7 | 3 | 2 | EUT1, PDU1A |
| 220341 | Gammaproteobacteria | *Salmonella enterica* subsp. *enterica* serovar Typhi str. CT18 | 2 | 0 | 0 | 7 | 4 | 1 | EUT1, PDU1A |
| 1132507 | Gammaproteobacteria | *Salmonella enterica* subsp. *enterica* serovar Typhi str. P-stx-12 | 2 | 0 | 0 | 7 | 3 | 2 | EUT1, PDU1A |
| 209261 | Gammaproteobacteria | *Salmonella enterica* subsp. *enterica* serovar Typhi str. Ty2 | 2 | 0 | 0 | 7 | 3 | 1 | EUT1, PDU1A |
| 527001 | Gammaproteobacteria | *Salmonella enterica* subsp. *enterica* serovar Typhi str. Ty21a | 2 | 0 | 0 | 6 | 3 | 2 | EUT1, PDU1A |
| 588858 | Gammaproteobacteria | *Salmonella enterica* subsp. *enterica* serovar Typhimurium str. 14028S | 2 | 0 | 0 | 7 | 3 | 2 | EUT1, PDU1A |
| 568708 | Gammaproteobacteria | *Salmonella enterica* subsp. *enterica* serovar Typhimurium str. D23580 | 2 | 0 | 0 | 6 | 3 | 2 | EUT1, PDU1A |
| 99287 | Gammaproteobacteria | *Salmonella enterica* subsp. *enterica* serovar Typhimurium str. LT2 | 2 | 0 | 0 | 7 | 3 | 2 | EUT1, PDU1A |
| 216597 | Gammaproteobacteria | *Salmonella enterica* subsp. *enterica* serovar Typhimurium str. SL1344 | 2 | 0 | 0 | 7 | 3 | 2 | EUT1, PDU1A |
| 990282 | Gammaproteobacteria | *Salmonella enterica* subsp. *enterica* serovar Typhimurium str. UK-1 | 2 | 0 | 0 | 7 | 3 | 2 | EUT1, PDU1A |
| 936157 | Gammaproteobacteria | *Salmonella enterica* subsp. *enterica* serovar Weltevreden str. 2007-60-3289-1 | 2 | 0 | 0 | 7 | 3 | 2 | EUT1, PDU1A |
| 1249634 | Gammaproteobacteria | *Serratia marcescens* FGI94 | 1 | 0 | 0 | 4 | 0 | 1 | GRM2 |
| 319224 | Gammaproteobacteria | *Shewanella putrefaciens* CN-32 | 1 | 0 | 0 | 3 | 1 | 1 | GRM4 |
| 351745 | Gammaproteobacteria | *Shewanella* sp. W3-18-1 | 1 | 0 | 0 | 3 | 1 | 1 | GRM4 |
| 344609 | Gammaproteobacteria | *Shigella boydii* CDC 3083-94 | 1 | 0 | 0 | 3 | 1 | 1 | EUT1 |
| 300268 | Gammaproteobacteria | *Shigella boydii* Sb227 | 1 | 0 | 0 | 3 | 1 | 1 | EUT1 |
| 300267 | Gammaproteobacteria | *Shigella dysenteriae* Sd197 | 1 | 0 | 0 | 2 | 1 | 0 | EUT1 |
| 591020 | Gammaproteobacteria | *Shigella flexneri* 2002017 | 1 | 0 | 0 | 2 | 1 | 0 | EUT1 |
| 198215 | Gammaproteobacteria | *Shigella flexneri* 2a str. 2457T | 1 | 0 | 0 | 2 | 1 | 0 | EUT1 |
| 198214 | Gammaproteobacteria | *Shigella flexneri* 2a str. 301 | 1 | 0 | 0 | 2 | 1 | 0 | EUT1 |
| 216599 | Gammaproteobacteria | *Shigella sonnei* 53G | 2 | 0 | 0 | 7 | 2 | 2 | EUT1, PDU1B |
| 300269 | Gammaproteobacteria | *Shigella sonnei* Ss046 | 2 | 0 | 0 | 7 | 3 | 2 | EUT1, PDU1B |
| 630626 | Gammaproteobacteria | *Shimwellia blattae* DSM 4481 = NBRC 105725 | 1 | 0 | 0 | 4 | 0 | 1 | GRM2 |
| 343509 | Gammaproteobacteria | *Sodalis glossinidius* str. 'morsitans' | 2 | 1 | 1 | 0 | 1 | 1 | all satellite-like |
| 717773 | Gammaproteobacteria | *Thioalkalimicrobium cyclicum* ALM1 | 1 | 0 | 0 | 2 | 1 | 2 | Alpha-Carboxysome |
| 1255043 | Gammaproteobacteria | *Thioalkalivibrio nitratireducens* DSM 14787 | 2 | 1 | 0 | 3 | 1 | 2 | Alpha-Carboxysome |
| 396595 | Gammaproteobacteria | *Thioalkalivibrio* sp. K90mix | 2 | 1 | 0 | 3 | 1 | 2 | Alpha-Carboxysome |
| 396588 | Gammaproteobacteria | *Thioalkalivibrio sulfidophilus* HL-EbGr7 | 2 | 1 | 0 | 3 | 1 | 2 | Alpha-Carboxysome |
| 765912 | Gammaproteobacteria | *Thioflavicoccus mobilis* 8321 | 1 | 0 | 0 | 2 | 1 | 2 | Alpha-Carboxysome |
| 317025 | Gammaproteobacteria | *Thiomicrospira crunogena* XCL-2 | 1 | 0 | 0 | 3 | 1 | 2 | Alpha-Carboxysome |
| 595494 | Gammaproteobacteria | *Tolumonas auensis* DSM 9187 | 1 | 0 | 0 | 4 | 2 | 1 | PDU1A |
| 903510 | Gammaproteobacteria | *Vibrio furnissii* NCTC 11218 | 1 | 0 | 0 | 4 | 0 | 1 | GRM2 |
| 1116375 | Gammaproteobacteria | *Vibrio* sp. EJY3 | 1 | 0 | 0 | 3 | 2 | 1 | GRM3 |
| 1262467 | Gammaproteobacteria | *Yersinia enterocolitica* (type O:2) str. YE3094/96 | 1 | 0 | 0 | 4 | 2 | 1 | PDU1A |
| 1262464 | Gammaproteobacteria | *Yersinia enterocolitica* (type O:9) str. YE56/03 | 1 | 0 | 0 | 4 | 2 | 1 | PDU1A |
| 393305 | Gammaproteobacteria | *Yersinia enterocolitica* subsp. *enterocolitica* 8081 | 1 | 0 | 0 | 4 | 2 | 1 | PDU1A |
| 994476 | Gammaproteobacteria | *Yersinia enterocolitica* subsp. *palearctica* 105.5R(r) | 1 | 0 | 0 | 4 | 2 | 1 | PDU1A |
| 930944 | Gammaproteobacteria | *Yersinia enterocolitica* subsp. *palearctica* Y11 | 1 | 0 | 0 | 4 | 2 | 1 | PDU1A |
|  |  |  |  |  |  |  |  |  |  |
| 439235 | Deltaproteobacteria | *Desulfatibacillum alkenivorans* AK-01 | 1 | 0 | 0 | 2 | 3 | 1 | PDU1 (unclustered) |
| 177439 | Deltaproteobacteria | *Desulfotalea psychrophila* LSv54 | 2 | 0 | 0 | 7 | 4 | 2 | GRM1 |
| 207559 | Deltaproteobacteria | *Desulfovibrio alaskensis* G20 | 1 | 0 | 0 | 4 | 3 | 1 | GRM1 |
| 525146 | Deltaproteobacteria | *Desulfovibrio desulfuricans* subsp. *desulfuricans* str. ATCC 27774 | 1 | 0 | 0 | 4 | 3 | 1 | GRM1 |
| 1121451 | Deltaproteobacteria | *Desulfovibrio hydrothermalis* AM13 = DSM 14728 | 1 | 0 | 0 | 4 | 3 | 1 | GRM1 |
| 526222 | Deltaproteobacteria | *Desulfovibrio salexigens* DSM 2638 | 1 | 0 | 0 | 4 | 3 | 1 | GRM1 |
| 502025 | Deltaproteobacteria | *Haliangium ochraceum* DSM 14365 | 3 | 1 | 1 | 1 | 3 | 3 | MIC (unclustered) |
|  |  |  |  |  |  |  |  |  |  |
| 1045858 | Spirochaetes | *Brachyspira intermedia* PWS/A | 1 | 0 | 0 | 5 | 2 | 1 | PDU1 (unclustered) |
|  |  |  |  |  |  |  |  |  |  |
| 891968 | Synergistetes | *Anaerobaculum mobile* DSM 13181 | 1 | 0 | 0 | 4 | 2 | 1 | PDU1 (unclustered) |
| 651822 | Synergistetes | *Synergistetes bacterium* SGP1 | 1 | 0 | 0 | 2 | 1 | 1 | MIC (unclustered) |
| 525903 | Synergistetes | *Thermanaerovibrio acidaminovorans* DSM 6589 | 1 | 0 | 0 | 3 | 2 | 1 | EUT2B |
| 580340 | Synergistetes | *Thermovirga lienii* DSM 17291 | 1 | 0 | 0 | 5 | 2 | 1 | PDU1 (unclustered) |
|  |  |  |  |  |  |  |  |  |  |
| 452637 | Verrucomicrobia | *Opitutus terrae* PB90-1 | 2 | 1 | 0 | 3 | 1 | 3 | PVM |
