# Supplementary material for: A Taxonomy of Bacterial Microcompartment Loci Constructed by a Novel Scoring Method
Source: PLoS Comput Biol. 2014 Oct 23;10(10):e1003898. doi: 10.1371/journal.pcbi.1003898 (PMC4207490; doi:10.1371/journal.pcbi.1003898)
Supplement: Text S1 — Genes and pfams associated with the PDU and EUT loci. (DOCX) [file pcbi.1003898.s012.docx]

**Supporting Text S1: Genes and pfams associated with the PDU and EUT loci.**

**Genes and Pfams Associated with the PDU Loci**

The PDU1A representative locus consists of homologs to the *pduCDEGHLOPQSVWX* genes (Fig. S2), as well as *pduF* and the regulatory gene *pocR* (PF12833, PF10114, PF00165). PduC (PF02286), PduD (PF02288), and PduE (PF02287) are the large, medium, and small subunits of the cobalamin-dependant propanediol dehydratase, PduP (PF00171) is the aldehyde dehydrogenase (AldDH), PduL (PF06130) is a phosphotransacetylase (PTAC), PduQ (PF00465) is an alcohol dehydrogenase (AlcDH), and PduW (PF00871) is a propionate kinase [1]. PduV (PF10662) is a protein of unknown function which has been implicated in spatial orientation of the PDU microcompartment in the cytoplasm [2]. PduF (PF00230) is a proposed propanediol diffusion facilitator [3]. Four genes within the PDU1A loci are related to cobalamin recycling: propanediol dehydratase reactivase PduGH (PF08841; PF02288) [4], ATP:cobalamin adenosyltransferase PduO (PF01923, PF03928) [5], and cobalamin reductase PduS (PF01512, PF13534, PF13375, PF10531). Additionally, PduX (PF00288) is a threonine kinase involved in cobalamin synthesis [6].

Within nearly all PDU1A loci but upstream of the *pdu* operon on the opposite strand are encoded the genes *cbiABCDEFGHT* (PF01656, PF07685; PF03186; PF02570; PF01888; PF00590; PF12847; PF11760, PF11761, PF01890) (Fig. S2, Dataset S1) whose gene products participate in the synthesis of cobinamide, a crucial first step in the synthesis of cobalamin [7]. These cobalamin synthesis genes correspond to the *cob* operon involved in cobalamin biosynthesis. In addition, a homolog to the aminotransferase CobD (PF00155) is frequently found immediately downstream of the *pdu* operon in PDU1A, although in the case of *Tolumonas auensis* DSM 9187 and the loci from the *Yersinia* genus, this protein is encoded within the putative *pdu* operon along with an additional cobalt-precorrin methyltransferase (PF00590).

A number of genes are conserved within the PDU1A locus in over half of the PDU1A loci: a *yeeX*-like protein of unknown function (PF04363: DUF496), a *yeeA*-like putative membrane transporter protein (PF04632), a *yeeB*-like small molecular binding domain protein (PF06445), and a *yeeC*/*dacD*-like D-alanyl-D-alanine carboxypeptidase (PF13354, PF07943, PF00768) which is involved in β-lactam antibiotic resistance [8] (Dataset S1). Also found in *Salmonella* are conserved the genes *phsABC* (PF04879, PF00384, PF01568; PF13237, PF13247, PF12797, PF12837, PF12838, PF13183, PF13187; PF01292) which encode a thiosulfate reductase, which reduces thiosulfate to hydrogen sulfide. Thiosulfate is produced when reducing tetrathionate, which serves as an electron acceptor when *S. enterica* is grown anaerobically on propanediol [9].

PDU1B loci are found in the genomes of *Escherichia coli* E24377A, *Escherichia coli* LF82, *Escherichia coli* UMN026, *Shigella sonnei* 53G, and *Shigella sonnei* Ss046 (Table S2). All PDU1B loci contain homologs to the *pdu* genes in the PDU1A locus except for PduX and PduW. The PDU1B locus encodes homologs to *cobSTU* (PF02654; PF02277; PF02283) cobalamin biosynthesis genes from the 3’ end of the *cob* operon which are responsible for joining adenosyl-cobinamide with 5,6-dimethylbenzimidazole to form adenosyl-cobalamin (vitamin B_12_) [10]. However, the remainder of the genes in the *cob* operon are not co-localized with the PDU1B loci. Also within the PDU locus are a *ykuD*-like peptidoglycan transpeptidase domain-containing protein (PF03734) and two *lysR*-type regulatory proteins (PF00126, PF03466) (Dataset S1). YkuD has been shown in *Enterococcus faecium* to confer high-level β-lactam resistance [11]. PDU1B loci contain the same *yeeXAC* genes as in PDU1A, as well as several more: homologs to *sbcB* exodeoxyribonuclease (PF00929, PF08411), *yeeF* membrane transporter (PF00324), *lysR* regulatory gene, and *yeeZ*, a putative epimerase (PF04363) (Dataset S1).

All PDU1C loci are found in three strains of *Lactobacillus reuteri* and as such have extremely high synteny in neighboring genes. PDU1C loci lack genes encoding PduS, PduX, and PocR, and the two domains in the PduO protein are encoded by two adjacent genes. Also encoded in PDU1C loci are homologs to PduF, the EutJ putative chaperone (PF00012, PF06723, PF11104), an AraC type regulatory protein (PF12833), a tyrosine phosphatase (PF13350, PF13348), a transporter associated with cadmium resistance (PF03596), a flavodoxin protein (PF00258), an NADPH-dependent FMN reductase (PF13508, PF14542, PF03358), and genes from the *cob* operon*.*

PDU1D loci are found in the genomes *Thermoanaerobacter* sp. X513 and *Thermoanaerobacter* sp. X513. PduX, PduS, and PocR are absent from PDU1D loci. This locus is bisected by a transposase gene (PF05598, PF01609) and additionally encodes a transmembrane protein of unknown function (PF02659), a flavoprotein (PF02441), a EutJ-like protein (PF11104), a PocR domain-containing histidine kinase (PF02518, PF06580, PF10114) and response regulator (PF12833, PF00072, PF00165), a homolog to the YtaF membrane-bound protein (PF02659: DUF204) [12], and an integrase domain protein (PF13936, PF00665, PF13384, PF13518). The *cob* operon is not found within PDU1D loci.

21 PDU1 loci did not cluster with any of these sub-types (Fig. S1), representing subtle modifications to the PDU1 locus. The three PDU1 loci from *Anaerobaculum mobile* DSM 13181, *Thermoanaerobacterium saccharolyticum* JW/SL-YS485, and *Thermoanaerobacterium xylanolyticum* LX-11 contain a class II aldolase (PF00596) in addition to the diol dehydratase (Dataset S1), which could be involved in the conversion of L-fucose/L-rhamnose to propanediol as has been recently proposed in a different locus [13]. Among PDU1 locus variants is one identified in *Escherichia fergusonii* ATCC 35469 that encodes a glycyl radical enzyme (GRE; PF01228, PF02901) and appears to be a fusion of the PDU1B and GRM2 loci (Dataset S1).

The PDU-like/PDU3 locus type appears to be a reduced PDU locus found only in *Clostridium kluyveri,* and of the canonical PDU genes, this locus only contains *pduCELSX*. Conspicuously, *pduD*, the gene encoding the medium subunit of the diol dehydratase, is not found anywhere in the entire genome. Neither an AldDH nor AlcDH are found in this locus, although a PduL PTAC is present. In addition, the diol dehydratase reactivating factor *pduGH* is missing from the locus. Interestingly, the 447 nucleotide long intergenic region immediately downstream of *pduE* bears weak homology to a 171 nucleotide region of *pduH* the reactivating factor from *Clostridium carboxidivorans* P7, indicating a possible incomplete deletion of the *pduH* gene from the locus. Another unique feature is the presence of eight genes encoding four PduS and four PduT BMC-T homologs. The first and second PduS homologs are 100% identical to the third and fourth PduS homologs, respectively, and the same trend holds for the PduT homologs, indicating a recent duplication of a segment of the locus containing two genes each alternating PduS and PduT homologs. Also within the locus are encoded a EutJ-like protein, a flavoprotein, four proteins containing iron-sulfur cluster domains, one of which bears homology to an iron hydrogenase (PF02256, PF02906, PF13237, PF13459), an ACT domain-containing protein (PF13291), and homologs to homoserine dehydrogenase (PF03435, PF00742, PF03447), homoserine kinase (PF00288, PF08544), and an acyl-homoserinelactone synthase (PF13420).

**Genes and Pfams Associated with the EUT Loci**

The vast majority of EUT1 loci contain homologs to the genes *eutABCDEGHJPQRT*, many of which have been well studied. EutB (PF06751) and EutC (PF05985) are the large and small subunits of ethanolamine ammonia lyase, EutE is the AldDH that converts acetaldehyde to acetyl-CoA [14], EutD (PF01515) is a *pta* PTAC of a different type than PduL that converts acetyl-CoA to acetylphosphate [15], and EutG is an AlcDH that metabolizes acetaldehyde to ethanol [16]. EutA (PF06277) is a reactivating factor for the ethanolamine ammonia lyase [17]. EutH (PF04346) encodes an ethanolamine facilitator that transports ethanolamine [18]. EutJ (PF11104), EutP (PF10662), and EutQ (PF06249) are proteins whose function in the EUT locus is not yet clear [19], though EutJ has been proposed to be a chaperone [20], and a homolog of EutP, PduV, in the PDU locus is thought to play a role in spatial orientation of the PDU microcompartment [2]. EutT encodes an ATP:cobalamin adenosyltransferase homologous to PduO [21].

All EUT1 loci save for one are found in members of the Gammaproteobacteria, the majority of them being in pathogenic enterobacteria from the genera *Enterobacter*, *Enterobacteriaceae*, *Escherichia*, *Citrobacter*, *Klebsiella*, *Salmonella*, and *Shigella*. These loci share much in common with *S. enterica*, resulting in a very tight network. Several non-pathogenic bacteria also contain a variation of this type: *Oleispira antarctica* RB-8 is an oil-degrading marine bacterium isolated from Antarctic sea water [22], *Morganella morganii* subsp. morganii KT is a commensal pathogen often found in intestinal infections [23], *Marinobacter aquaeolei* VT8 is a halophile isolated from an off-shore oil well in Vietnam [24], and *Anaerolinea thermophila* UNI-1 from the phylum Chloroflexi was isolated from thermophilic anaerobic granular sludge [25]. The latter three are clear outliers within the EUT1 locus similarity network (Fig. S1): *M. aquaeolei* and *M. morganii* contain all the genes from the *S. enterica* *eut* operon in their loci, as well as a PduS homolog; however, the genes outside of the operon conserved in other EUT1 loci are not present in these loci (Dataset S1). *A. thermophila*’s EUT1 locus is missing the protein of unknown function EutP, as well as the AlcDH and PTAC genes; this locus is therefore likely not functionally complete. In addition, only *A. thermophila* and *M. aquaeolei* do not encode the *eutR* regulatory gene in the EUT1 locus (Dataset S1).

Also encoded within EUT1 loci are the MaeB malic enzyme (PF00390, PF03949, PF01515) that catalyzes the conversion of malate to pyruvate with the release of CO_2_, a TalA-like putative transaldolase (PF00923), a putative transketolase (PF02779, PF00456, PF02780), a domain of unknown function (PF06674: DUF1176), a NUDIX domain protein (PF00293), a HemF coproporphyrinogen III oxidase (PF01218), a YfeX porphyrinogen oxidase (PF04261) [26], a putative N-acetylmuramoylalanine amidase (PF01520), a putative N-acetyltransferase (PF00583), a putative inner membrane protein (PF11143: DUF2919), and a protein of unknown function (PF06752: DUF1131).

While most of the EUT1 loci contain the EUT signature enzyme genes, a number of these loci do not. Of the eight *Shigella* EUT1 loci, four are either missing or contain only a pseudogene copy of one of the ethanolamine ammonia-lyase proteins, while three are also missing the e*utA*. The *Escherichia coli* IAI39 locus also contains only a *eutB* pseudogene fragment. In addition, the *Escherichia coli* str. K-12 substr. MG1655 locus contains a phage integrase (PF00589, PF14659, PF13356: DUF4102) followed by five copies of the CPZ-44 prophage predicted protein. It is possible that these loci are in the process of losing the capability to utilize ethanolamine.

The majority of EUT2 loci contain *eutABCEHPQ*. Instead of the EutD PTAC, these loci usually encode a homolog to the PduL PTAC (PF06130). In place of the EutR regulatory enzyme found in EUT1 loci, a two-component regulatory system consisting of a signal transduction histidine kinase (PF02518, PF07568, PF13581, PF12282, PF13426) and a response regulator (PF00072 and PF03861) tend to be found in EUT2 loci.

EUT2A’s most defining feature is the *eutG* gene encoding an AlcDH necessary for cofactor recycling, which is absent from most other EUT2 loci. One or more copies of a transporter protein (PF00005) are encoded in approximately half of the EUT2 loci.

Over half of EUT2B loci additionally encode a homolog to the EutJ putative chaperone. Uniquely, only EUT2B and EUT2D sub-types contain a gene encoding a homolog of PduS cobalamin reductase [27,28].

While EUT2C loci do not encode AlcDH, they do encode lactate/malate dehydrogenase homolog (PF03721, PF00056, PF02866). Since lactate/malate dehydrogenase is an oxidoreductase, it is possible that this enzyme can perform the same function as AlcDH, though experimental evidence is necessary to test this hypothesis.

The EUT2D locus is unique to the genomes of *Desulfosporosinus meridiei* DSM 13257 and *Thermincola potens* JR. While most of the same genes are found within and flanking the EUT2D locus, there is a low degree of synteny between the two loci. In addition, the locus of *T. potens* appears to be an expanded form of the locus, uniquely among these two loci encoding homologs to EutJ, EutP, EutT, and PduL. These loci encode homologs to PduS, as well as homoserine kinase (PF01636) and a trimethylamine methyltransferase homolog (PF06253).

The EUT3 locus type represents an extremely reduced EUT locus. Of the canonical EUT genes, only *eutABCPT* are encoded in the locus. Also encoded are a LysR-like regulatory protein (PF00126 and PF03466), a trimethylamine methyltransferase homolog, similar to that found in EUT2D, and a phosphodiesterase (PF01663).

A number of individual EUT loci either could not be clustered into EUT1 or EUT2 types or appeared in entirely distinct clusters. The former include the actinobacterium *Nocardioides* sp. JS614 and the firmicutes *Coprococcus catus* GD/7 and *Halanaerobium hydrogeniformans*. These represent additional possibly incomplete variations on the EUT locus. None of these encode AlcDH or either PTAC gene found in EUT loci, and only *Nocardioides* encodes an AldDH. Furthermore, while *eutAC* is encoded in all three loci, *eutB* is absent from *C. catus*. In addition, several EUT loci appear outside of Cluster 3. The first of these from *Desulfosporosinus orientis* DSM 765 appears in Cluster 2. In Cluster 10, an extremely reduced EUT locus from *Symbiobacterium thermophilum* IAM 14863 encodes only a single BMC-H protein and EutAB.

**References**

1. Palacios S, Starai VJ, Escalante-Semerena JC (2003) Propionyl Coenzyme A Is a Common Intermediate in the 1,2-Propanediol and Propionate Catabolic Pathways Needed for Expression of the *prpBCDE* Operon during Growth of *Salmonella enterica* on 1,2-Propanediol. J Bacteriol 185: 2802–2810. Available: http://jb.asm.org/content/185/9/2802. Accessed 23 May 2014.

2. Parsons JB, Frank S, Bhella D, Liang M, Prentice MB, et al. (2010) Synthesis of empty bacterial microcompartments, directed organelle protein incorporation, and evidence of filament-associated organelle movement. Mol Cell 38: 305–315. Available: http://www.ncbi.nlm.nih.gov/pubmed/20417607. Accessed 23 May 2014.

3. Chen P, Andersson DI, Roth JR (1994) The control region of the *pdu/cob* regulon in *Salmonella typhimurium*. J Bacteriol 176: 5474–5482. Available: http://jb.asm.org/content/176/17/5474. Accessed 6 March 2014.

4. Walter D, Ailion M, Roth J (1997) Genetic characterization of the *pdu* operon: use of 1,2-propanediol in *Salmonella typhimurium*. J Bacteriol 179: 1013–1022. Available: http://www.ncbi.nlm.nih.gov/pmc/articles/PMC178792/. Accessed 6 March 2014.

5. Johnson CL V, Pechonick E, Park SD, Havemann GD, Leal NA, et al. (2001) Functional genomic, biochemical, and genetic characterization of the *Salmonella pduO* gene, an ATP:cob(I)alamin adenosyltransferase gene. J Bacteriol 183: 1577–1584. Available: http://jb.asm.org/content/183/5/1577. Accessed 23 May 2014.

6. Fan C, Bobik TA (2008) The PduX enzyme of *Salmonella enterica* is an L-threonine kinase used for coenzyme B12 synthesis. J Biol Chem 283: 11322–11329. Available: http://www.jbc.org/content/283/17/11322. Accessed 6 March 2014.

7. Chen P, Ailion M, Weyand N, Roth J (1995) The end of the cob operon: evidence that the last gene (cobT) catalyzes synthesis of the lower ligand of vitamin B12, dimethylbenzimidazole. J Bacteriol 177: 1461–1469. Available: http://jb.asm.org/content/177/6/1461. Accessed 21 February 2014.

8. Sarkar SK, Dutta M, Chowdhury C, Kumar A, Ghosh AS (2011) PBP5, PBP6 and DacD play different roles in intrinsic β-lactam resistance of *Escherichia coli*. Microbiology 157: 2702–2707. Available: http://mic.sgmjournals.org/content/157/9/2702. Accessed 6 March 2014.

9. Price-Carter M, Tingey J, Bobik TA, Roth JR (2001) The alternative electron acceptor tetrathionate supports B12-dependent anaerobic growth of *Salmonella enterica* serovar typhimurium on ethanolamine or 1,2-propanediol. J Bacteriol 183: 2463–2475. Available: http://www.ncbi.nlm.nih.gov/pmc/articles/PMC95162/. Accessed 6 March 2014.

10. Escalante-Semerena JC, Johnson MG, Roth JR (1992) The CobII and CobIII regions of the cobalamin (vitamin B12) biosynthetic operon of *Salmonella typhimurium*. J Bacteriol 174: 24–29. Available: http://www.pubmedcentral.nih.gov/articlerender.fcgi?artid=205671&tool=pmcentrez&rendertype=abstract. Accessed 6 March 2014.

11. Mainardi JL, Legrand R, Arthur M, Schoot B, van Heijenoort J, et al. (2000) Novel mechanism of β-lactam resistance due to bypass of DD-transpeptidation in *Enterococcus faecium*. J Biol Chem 275: 16490–16496. Available: http://www.jbc.org/content/275/22/16490. Accessed 6 March 2014.

12. Abecasis AB, Serrano M, Alves R, Quintais L, Pereira-Leal JB, et al. (2013) A genomic signature and the identification of new sporulation genes. J Bacteriol 195: 2101–2115. Available: http://jb.asm.org/content/195/9/2101.full. Accessed 25 February 2014.

13. Petit E, LaTouf WG, Coppi M V, Warnick TA, Currie D, et al. (2013) Involvement of a Bacterial Microcompartment in the Metabolism of Fucose and Rhamnose by *Clostridium phytofermentans*. PLoS One 8: e54337. Available: http://www.pubmedcentral.nih.gov/articlerender.fcgi?artid=3557285&tool=pmcentrez&rendertype=abstract. Accessed 28 February 2013.

14. Roof DM, Roth JR (1989) Functions required for vitamin B12-dependent ethanolamine utilization in *Salmonella typhimurium*. J Bacteriol 171: 3316–3323. Available: http://jb.asm.org/content/171/6/3316. Accessed 4 March 2014.

15. Brinsmade SR, Escalante-Semerena JC (2004) The *eutD* Gene of *Salmonella enterica* Encodes a Protein with Phosphotransacetylase Enzyme Activity. J Bacteriol 186: 1890–1892. Available: http://jb.asm.org/content/186/6/1890. Accessed 23 May 2014.

16. Kofoid E, Rappleye C, Stojiljkovic I, Roth J (1999) The 17-Gene Ethanolamine (*eut*) Operon of *Salmonella typhimurium* Encodes Five Homologues of Carboxysome Shell Proteins. J Bacteriol 181: 5317–5329. Available: http://www.ncbi.nlm.nih.gov/pmc/articles/PMC94038/.

17. Mori K, Bando R, Hieda N, Toraya T (2004) Identification of a reactivating factor for adenosylcobalamin-dependent ethanolamine ammonia lyase. J Bacteriol 186: 6845–6854. Available: http://jb.asm.org/content/186/20/6845.long. Accessed 4 March 2014.

18. Penrod JT, Mace CC, Roth JR (2004) A pH-sensitive function and phenotype: evidence that EutH facilitates diffusion of uncharged ethanolamine in *Salmonella enterica*. J Bacteriol 186: 6885–6890. Available: http://jb.asm.org/content/186/20/6885. Accessed 4 March 2014.

19. Held M, Quin MB, Schmidt-Dannert C (2013) Eut bacterial microcompartments: insights into their function, structure, and bioengineering applications. J Mol Microbiol Biotechnol 23: 308–320. Available: http://www.karger.com/Article/FullText/351343. Accessed 8 August 2013.

20. Stojiljkovic I, Bäumler AJ, Heffron F (1995) Ethanolamine utilization in *Salmonella typhimurium*: nucleotide sequence, protein expression, and mutational analysis of the *cchA cchB eutE eutJ eutG eutH* gene cluster. J Bacteriol 177: 1357–1366.

21. Buan NR, Suh S-J, Escalante-Semerena JC (2004) The *eutT* gene of *Salmonella enterica* Encodes an oxygen-labile, metal-containing ATP:corrinoid adenosyltransferase enzyme. J Bacteriol 186: 5708–5714. Available: http://jb.asm.org/content/186/17/5708. Accessed 4 March 2014.

22. Yakimov MM, Giuliano L, Gentile G, Crisafi E, Chernikova TN, et al. (2003) *Oleispira antarctica* gen. nov., sp. nov., a novel hydrocarbonoclastic marine bacterium isolated from Antarctic coastal sea water. Int J Syst Evol Microbiol 53: 779–785. Available: http://www.ncbi.nlm.nih.gov/pubmed/12807200. Accessed 11 February 2014.

23. Falagas ME, Kavvadia PK, Mantadakis E, Kofteridis DP, Bliziotis IA, et al. (2006) *Morganella morganii* infections in a general tertiary hospital. Infection 34: 315–321. Available: http://www.ncbi.nlm.nih.gov/pubmed/17180585. Accessed 11 February 2014.

24. Huu NB, Denner EB, Ha DT, Wanner G, Stan-Lotter H (1999) *Marinobacter aquaeolei* sp. nov., a halophilic bacterium isolated from a Vietnamese oil-producing well. Int J Syst Bacteriol 49: 367–375. Available: http://www.ncbi.nlm.nih.gov/pubmed/10319457. Accessed 11 February 2014.

25. Sekiguchi Y, Yamada T, Hanada S, Ohashi A, Harada H, et al. (2003) *Anaerolinea thermophila* gen. nov., sp. nov. and *Caldilinea aerophila* gen. nov., sp. nov., novel filamentous thermophiles that represent a previously uncultured lineage of the domain *Bacteria* at the subphylum level. Int J Syst Evol Microbiol 53: 1843–1851. Available: http://www.ncbi.nlm.nih.gov/pubmed/14657113. Accessed 11 February 2014.

26. Dailey HA, Septer AN, Daugherty L, Thames D, Gerdes S, et al. (2011) The *Escherichia coli* protein YfeX functions as a porphyrinogen oxidase, not a heme dechelatase. MBio 2: e00248–11. Available: http://mbio.asm.org/content/2/6/e00248-11. Accessed 3 March 2014.

27. Cheng S, Bobik TA (2010) Characterization of the PduS cobalamin reductase of *Salmonella enterica* and its role in the Pdu microcompartment. J Bacteriol 192: 5071–5080. Available: http://www.ncbi.nlm.nih.gov/pmc/articles/PMC2944522. Accessed 23 May 2014.

28. Parsons JB, Lawrence AD, McLean KJ, Munro AW, Rigby SEJ, et al. (2010) Characterisation of PduS, the *pdu* metabolosome corrin reductase, and evidence of substructural organisation within the bacterial microcompartment. PLoS One 5: e14009. Available: http://www.ncbi.nlm.nih.gov/pmc/articles/PMC2982820/. Accessed 23 May 2014.
